# North-Western Himalayan Common Beans: Population Structure and Mapping of Quantitative Anthracnose Resistance Through Genome Wide Association Study

**DOI:** 10.3389/fpls.2020.571618

**Published:** 2020-10-06

**Authors:** Aqleema Banoo, Asha Nabi, Rovidha S. Rasool, Mahiya-Farooq, Mehraj D. Shah, Mushtaq Ahmad, Parvaze A. Sofi, Aasiya-Nabi, Hamidullah Itoo, P. N. Sharma, Bilal A. Padder

**Affiliations:** ^1^Plant Virology and Molecular Plant Pathology Laboratory, Division of Plant Pathology, SKUAST-Kashmir, Srinagar, India; ^2^Directorate of Extension, SKUAST-Kashmir, Srinagar, India; ^3^Division of Genetics and Plant Breeding, Faculty of Agriculture, SKUAST-Kashmir, Wadura, India; ^4^Ambri Apple Research Centre, SKUAST-Kashmir, Srinagar, India; ^5^Department of Plant Pathology, CSK HPKV, Palampur, India

**Keywords:** *Phaseolus vulgaris*, SNPs, genome wide association study, anthracnose resistance, quantitative trait loci, northwestern Himalayan beans

## Abstract

Common bean (*Phaseolus vulgaris* L.) is an important legume crop of north-western (NW) Himalayan region and the major disease that causes catastrophic loss to the crop is anthracnose, which is caused by *Colletotrichum lindemuthianum*. The pathogen is highly diverse and most of the commercial cultivars are susceptible to different races prevalent in the region. The lack of information on the genomic regions associated with anthracnose resistance in NW Himalayan common bean population prompted us to dissect Quantitative Resistance Loci (QRLs) against major anthracnose races. In this study, 188 common bean landraces collected from NW region were screened against five important anthracnose races and 113 bean genotypes showed resistance to one or multiple races. Genotyping by sequencing (GBS) was performed on a panel of 192 bean lines (4 controls plus 188 Indian beans) and 22,589 SNPs were obtained that are evenly distributed. Population structure analysis of 192 bean genotypes categorized 188 Indian beans into two major clusters representing Andean and Mesoamerican gene pools with obvious admixtures. Many QRLs associated with anthracnose resistance to Indian *C. lindemuthianum* virulences (race 3, 87, and 503) are located at Pv04 within the gene models that encode typical resistance gene signatures. The QRLs associated with race 73 are located on Pv08 and overlaps with *Co-4* anthracnose resistance gene. A SNP located at distal end of Pv11 in a gene model Phvul.011G202300 which encodes a LRR with a typical NB-ARC domain showed association with race 73 resistance. Common bean genomic regions located at Pv03, Pv09, and Pv11 showed association with resistance to anthracnose race 2047. The present study showed presence of many novel bean genomic regions associated with anthracnose resistance. The presence of *Co-4* and *Co-2* genes in our material is encouraging for breeding durable anthracnose resistant cultivars for the region.

## Introduction

By 2050, the world population may increase by 2 billion and the important challenge before the humanity is to achieve food security and livelihood especially in developing countries. There is a need to increase the productivity of cheaper yet nutritionally rich crops. In this backdrop, *Phaseolus vulgaris*, a grain legume crop is a key commodity because of its rich nutrient profile (Broughton et al., 2003). Further, the nitrogen fixing capacity of bean crop enhances the soil fertility besides reduces environmental hazards of fertilizer application (Beaver et al., 2003; Rispail et al., 2010; Kamfwa et al., 2019). *Phaseolus vulgaris* is native to Andean and Mesoamerican regions where independent domestication resulted into two distinct major gene pools (Singh et al., 1991; Bitocchi et al., 2013; Bellucci et al., 2014; Pathania et al., 2014). Countries such as China, Africa, Europe, and Latin America are considered as secondary centers of beans (Duran et al., 2005; Asfaw et al., 2009; Pathania et al., 2014).

Common bean is an important legume crop of north-western (NW) Himalayan region, the largest producer of dry beans in India (Sofi et al., 2014; Rana et al., 2015). The Himalayan region is rich in common bean biodiversity and there is a scarcity of information about its dissemination in India (Pathania et al., 2014; Sofi et al., 2014). Europeans and Chinese who travelled to India for trading brought bright colored beans with them (Joshi and Thomas, 1987; Joshi and Mehra, 1993). Besides natural selection, farmers in the region selected bean genotypes according to their adaptability, taste and agronomic features that further enhanced bean diversity (Pathania et al., 2014). Common beans in India are grown over an area of 9.1 million ha with production of 3.63 million tons and yield of 399.0 kg/ha (Rana et al., 2015; Choudhary et al., 2018). Being nutritionally rich, it is socially an important crop in the NW states of India (Tiwari et al., 2005; Rana et al., 2012; Rana et al., 2015). The landraces particularly grown in the mountainous regions fetch higher market price (Rai et al., 2010; Rana et al., 2012). There are many famous bean landraces in the NW part of India such as “Chamba”, “Barot”, “Kinnauree”, “Auli”, “Munsiyari”, “Harshi”, “Bhaderwah”, and “Kashmiri” (Rana et al., 2015).

Many fungal, bacterial, and viral diseases infect common beans in northern India, but bean anthracnose caused by the hemibiotrophic fungus, *Colletotrichum lindemuthianum*, is a serious disease of *P. vulgaris* globally (Pastor-Corrales and Tu, 1989; Kumar et al., 1999; Pathania et al., 2006; Sharma et al., 2007; Sharma et al., 2008; Padder et al., 2010; Oblessuc et al., 2012). The pathogen is seed borne and planting of infected seed causes 80 to 100% yield loss (Sharma et al., 2008; Padder et al., 2017). Farmers in northern India plant their own saved seed that predisposes the crop to seed borne anthracnose infection. Further, the majority of the cultivated areas in the NW region of India experience frequent and high intensity rainfall during the cropping season, providing favorable conditions for the disease development; hence, anthracnose reoccurs regularly (Sharma et al., 1994; Sharma et al., 1999; Pathania et al., 2006; Sharma et al., 2007; Padder et al., 2009; Sharma et al., 2012). The pathogen is highly variable and 45 races are present in the NW region (Sharma et al., 1999; Pathania et al., 2006; Padder et al., 2007; Sharma et al., 2007; Padder et al., 2009; Padder et al., 2010; Sharma et al., 2019). Most of these races are different from those in the United States, Brazil, France, Canada, and many other countries. Being pathogenically variable, majority of commercially grown cultivars are susceptible to one or the other race(s) of the pathogen (Sharma et al., 2000; Pathania et al., 2006; Sharma et al., 2006; Sharma et al., 2012) which necessities the identification of durable anthracnose resistance cultivars.

Systematic studies on identification and mapping of anthracnose resistance genes in the United States, Canada, Brazil, and France resulted in the identification of around 25 anthracnose resistance genes. Most of the anthracnose resistance genes are dominant except the *co-8* and are mapped to eight bean chromosomes (Burt et al., 2015; Meziadi et al., 2016; Zuiderveen et al., 2016). Seventeen of these genes are numbered from *Co-1* to *Co-17*, however many anthracnose resistance genes have alphabets as suffix such as *Co-x*, *Co-y*, *Co-w*, *Co-Pa*, *Co-z*, *Co-1^HY^*, and are mapped to bean chromosomes where numbered genes are located (Zuiderveen et al., 2016). *Co-1* and its alleles, *Co-14*, *Co-x*, *Co-w*, *Co-1^HY^*, *Co-1^65-X^*, *Co-1^73-X^*, and *Co-Pa* anthracnose resistance genes are mapped to the distal end of bean chromosome Pv01 (Melotto and Kelly, 2000; Geffroy et al., 2008; Gonçalves-Vidigal et al., 2011; Campa et al., 2014; Richard et al., 2014; Chen et al., 2017; de Lima Castro et al., 2017). Five anthracnose resistance genes (*Co-u*, *CoPv02c^3-X^*, *CoPv02c^7-X^*, *CoPv02c^19-X^*, and *CoPv0c^2449-X^*) are mapped to Pv02 (Kelly et al., 2003; Campa et al., 2014). Anthracnose resistance genes *Co-13* and *Co-17* are mapped to Pv03 (Goncalves-Vidigal et al., 2009; Lacanallo and Gonçalves-Vidigal, 2015; Trabanco et al., 2015). The anthracnose resistance gene mapped to Pv04 are *Co-3* and its alleles, *Co-10*, *Co-y*, *Co-z Co-15*, *Co-16*, and *Co-RVI* (Geffroy et al., 2008; Coelho et al., 2013; Goncalves-Vidigal et al., 2013; Sousa et al., 2015; Coimbra-Gonçalves et al., 2016). Anthracnose resistance genes *Co-5*, *Co-6*, and *Co-v* map to Pv07 (Campa et al., 2009). Anthracnose resistance genes *Co-4* and its alleles and *Co-2* are located on Pv08 and Pv11, respectively (Young et al., 1998; Melotto and Kelly, 2001; Alzate-Marin et al., 2007; Rodriguez-Suarez et al., 2007). In addition to major resistance genes, studies showed that anthracnose resistance is quantitatively (QTLs) inherited like ANT02.1^UC^ and ANT07.1^UC^ (Oblessuc et al., 2014). Recently, a major QTL for anthracnose resistance against race 1545 was mapped to Pv05 with candidate gene Phvul.005G117900 that encodes a NBS-LRR protein (Gonzalez et al., 2015). Genome wide association studies (GWAS) showed association of a SNP and SSR marker located at Pv10 with anthracnose resistance (Zuiderveen et al., 2016; Wu et al., 2017). Access to *P. vulgaris* whole genome (Schmutz et al., 2014; Vlasova et al., 2016) and SNP Bean Chips (Hyten et al., 2010; Song et al., 2015) has resulted in the fine mapping of many economically important traits in beans including anthracnose resistance (Richard et al., 2014; Burt et al., 2015; Gonzalez et al., 2015; Oblessuc et al., 2015; Padder et al., 2016; Zuiderveen et al., 2016; Chen et al., 2017; de Lima Castro et al., 2017; Mungalu et al., 2020).

In India, a few studies identified anthracnose resistant sources to Indian races (Sharma et al., 1993; Kumar et al., 1997; Sharma et al., 1999; Pathania et al., 2006; Sharma et al., 2006; Sharma et al., 2012). Among the various sources of resistance, only a few genotypes were exploited to know the genetics of resistance (Sharma et al., 2000; Pathania et al., 2006; Katoch et al., 2019). For instance, inheritance pattern of broad spectrum anthracnose resistant cultivars such as KRC-5 and Baspa (KRC-8) showed dominant and recessive anthracnose resistance genes, respectively to a range of anthracnose races (Sharma et al., 2000; Pathania et al., 2006). The high diversity in common bean coupled with resistance to anthracnose races warrants identification of genomic regions associated with anthracnose. Mapping anthracnose resistance genes *via* bi-parental mapping populations is time consuming thus GWAS is the easiest approach to identify the genomic regions associated with anthracnose resistance genes in NW Himalayan common beans. Like the USA and other countries, India also need to exploit GWAS to its full potential to discern different agronomic and resistance traits in the diverse bean germplasm. Here we report a SNP based population structure of 188 common beans collected from NW Himalayan region. In the present study, genotyping by sequencing (GBS) approach was used with the aim to infer the population structure, to identify common beans with broad-spectrum resistance to anthracnose disease and to identify common bean genomic regions associated with anthracnose resistance to major anthracnose races present in the region.

## Material and Methods

### Common Bean Genotype Collection and Bean Anthracnose Race Material

Common beans cultivated in the NW Himalayan region are highly diverse (Rana et al., 2015) and we selected 188 common bean lines that were collected from SKUAST-K, Wadura, Himachal Pradesh Krishi Vishvavidyala (HPKV), Palampur, National Bureau of Plant Genetic Resources (NBPGR) located at Shimla and New Delhi. Four genotypes such as AND 277, Kaboon (Andean genotypes), TO, and Michelite (Mesoamerican genotypes) were used as checks for population structure study. **Supplementary Table 1** contains the passport data of 192 lines that were used in the present study. All the bean genotypes were purified by a single seed decent method for three consecutive years.

We used five *C. lindemuthianum* races (3, 73, 87, 503, and 2047) to phenotype 188 bean genotype and choice of races was based on their virulence and geographic prevalence. Bean anthracnose race 87 is present in two states of NW India, whereas race 503 is a predominant across NW region (Sharma et al., 2019). Race 3 is present in major bean growing areas of Himachal Pradesh (Sharma et al., 2007). Anthracnose race 73 is an important race in the United States and Canada (Kelly et al., 1994; Rio et al., 2003a; Rio et al., 2003b; Conner et al., 2019). Race 2047 is highly virulent because it defeats most of the major anthracnose resistance genes (**Supplementary Table 2**) and only few genotypes show resistance against it (Zuiderveen et al., 2016). Professor James D. Kelly, Michigan State University, kindly provided race 73 and 2047. All *C. lindemuthianum* races are available in the Division of Plant Pathology, SKUAST-K, Shalimar and are maintained on sterilized Whatman filter paper discs and in glycerol stocks at -20°C for long time storage. To avoid virulence loss, races are inoculated to susceptible genotypes and pathogen is re-isolated from infected stem tissue.

### Phenotype Screening

**Figure 1:**
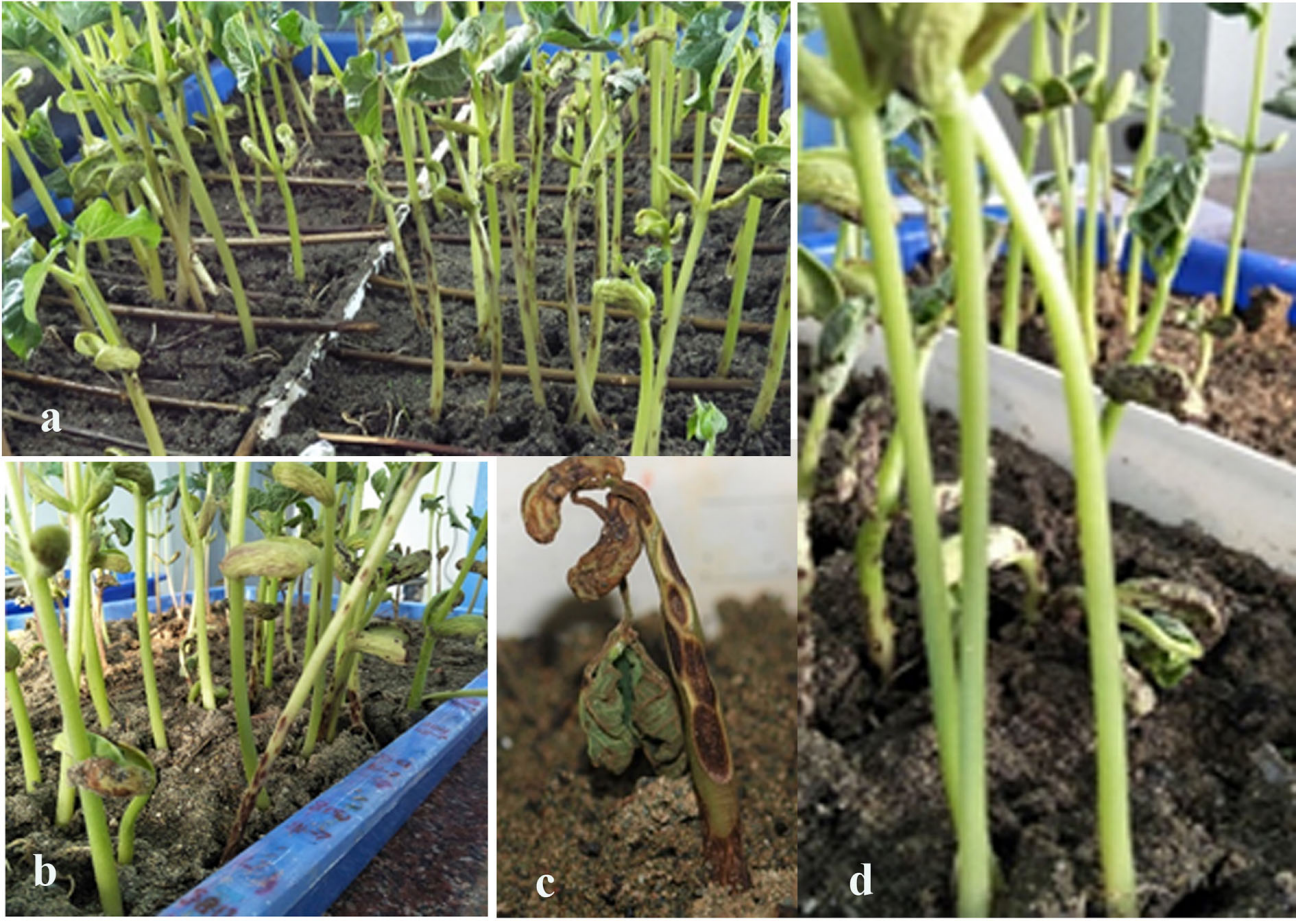
Screening of common bean population with anthracnose races **(a, b)**. Plants showing disease score 5 were killed **(c)**; whereas plants with 0 score showed no symptoms **(d)**.

For screening bean genotypes to anthracnose races, six seeds of each line were planted in a tray containing coco-peat and vermiculite (2:1 ratio). The cotyledonary leaves were sprayed with a spore suspension that was prepared from seven days old culture by scraping the surface of culture with a spatula and few drops of 0.01% Tween 20 were added in sterile distilled water. Inoculum load was adjusted to 2 × 10^6^ conidia/ml after counting the number of spores in an initial suspension with a haemocytometer. Inoculated plants were kept in plant growth chamber at 22°C with 90% humidity, 14:10-h day/night photoperiod, and light intensity of 120–150 µE/m^2^ (Mahiya-Farooq et al., 2019). The disease reaction was recorded with the 0–5 scale as described elsewhere (Drijfhout and Davis, 1989). Plants that were scored 0, 1, or 2 were categorized as resistant, whereas plants with scores of 3, 4, or 5 were graded as susceptible (**Figure 1**). The plant growth chamber was sterilized with 70% ethanol before performing screens of bean genotypes with other races. The phenotypic data was analyzed using R package and summary statistics such as density and frequency were calculated.

### Genotyping by Sequencing

For GBS, the total genomic DNA from 192 bean genotypes was extracted from young leaf tissues using CTAB (Hexadecyltrimethyl ammonium bromide) protocol as described by Murray and Thompson (1980). DNA integrity and quality was checked by Agarose gel electrophoresis and quantification was performed on an Eppendroff nano-bio-spectrophotometer. The DNA samples were diluted to 25 ng/ml by adding nuclease free water. Approximately, 30 μl of each DNA sample from all the genotypes were shipped to Xcelris Labs Limited, Gujarat, India for GBS.

### Preparation of GBS Library and Quantity/Quality Check

The 100-ng genomic DNA from 192 samples was digested using A*pe*K1 enzyme and the GBS library was prepared from the digested DNA fragments by ligating adaptors specific to the cut-site. The ligated products were pooled into 16 groups of 12 samples each. The pool was PCR amplified to generate the final library pool. The library pools were analyzed in Bioanalyzer 2100 (Agilent Technologies) using High Sensitivity (HS) DNA chip as per manufacturer’s instructions. Then next generation sequencing for all the samples was carried out independently on IlluminaHiSeq platform to generate up to 3.5–4.0 million reads.

### Bioinformatics Analysis

The *fastq* files obtained from 192 samples in 16 different pools were first de-multiplexed and then we calculated overall amount of data and performed the QC. The high quality (HQ) data was extracted using Trimmomatic at Q20 quality values. The barcode tagged *fastq* files for both R1 and R2 (paired end) were condensed into single file and analyzed using TASSEL. The Tassel key file containing the information of each barcode corresponding to each sample was generated. Common bean reference genome (*P. vulgaris* v2.0) was downloaded from Phytozome (https://genome.jgi.doe.gov/portal/pages/dynamicOrganismDownload.jsf?organism=Pvulgaris). The genome has 11 chromosomes and 467 scaffolds with 537.22-Mb genome size. All reads from each sample were aligned to the common bean reference genome (v2.0).

We used TASSEL5 GBSv2 pipeline for identification of tags at cut sites and SNPs located across reference genome. The GBSv2 pipeline supports *fastq* files in the older format, which includes multiple taxa combined in a single file with barcodes attached to each read sequence. The *ApekI* tags were determined using GBSSeqToTagDBPlugin, which identifies tags from *fastq* input files then stores these tags and the taxa in which they appear into a local SQLite database. Next TagExportToFastqPlugin with default parameters was used to pull distinct tags from the database that were exported in the *fastq* format so that they can be aligned to the reference genome with various aligners. The *fastq* files were aligned against the common bean reference genome v.2.0 downloaded from phytozome using the Bowtie2 tool version 2.2.9 (Langmead and Salzberg, 2012). The sam file created from the Bowtie2 aligner program was used through SAMToGBSdbPlugin to store the position information for each aligned tag. We then used DiscoverySNPCallerPluginV2 to identify SNPs from the aligned tags. SNP positions for each allele and the tags associated with that allele were exported along with the number of times the tag appears in each taxa. SNP coverage, quality and genotypic statistics were calculated using SNPQualityProfilerPlugin. Finally, ProductionSNPCallerPluginV2 plugin was used for SNP calling. To acquire high quality SNPs with minimum missing data, filtration criteria of missing data was applied with following parameters; minor allele frequency (MAF >= 0.05, MAC >= 10) using VCF tools as per the procedure described by Mourad et al. (2018).

### Population Structure, Kinship, and Linkage Decay (LD) Analysis

Structure threader software was used to assess the population genetic structure among 192 common bean (Pina-Martins et al., 2017). Population structure was estimated based on total SNP loci (22,589 SNPs) and K from 1 to 10 with 10 independent runs for each K. To determine the probable number of clusters based on genotypes we used 5,000 burnin and 50,000 MCMC (Markov Chain Monte Carlo) iteration. Structure output was then subjected to structure harvester for identification of effective number of clusters using the Evanno test implement in STRUCTURE HARVESTER (Earl, 2012). The principal component analysis was performed using TASSEL5.0 to determine the percentage of variation explained by top three principal components. For phylogenetic analysis, SNPhylo was run considering parameter of bootstrapping and multiple alignment by MUSCLE and the tree was plotted using figtree v1.4.1. To know the relatedness among 192 common bean genotypes based on shared alleles among individuals, we used GAPIT R package for generation of Kinship matrix. All the 22,589 filtered SNPs were used to perform the analysis because previous studies have shown that the complete set of markers control genome wide error rate better and is a good choice than pedigree based methods for kinship estimates. Linkage Disequilibrium (LD) was estimated using Genomic Association and Prediction Integrated Tool (GAPIT) and LD curve was fitted using a nonlinear model as described elsewhere (Marroni et al., 2011; Gao L. et al., 2016).

### Genome Wide Association Analysis

After removing four controls (Kaboon, AND 277, Michelite, and TO) and eight genotypes that were phenotypically heterozygous, 180 genotypes were processed for GWAS analysis. GAPIT was used for GWAS analyses and SNP-trait association analysis was performed using Mixed Linear Model (MLM) (Zhang et al., 2010) implemented in GAPIT that include correction for both population structure and kinship. The qqman R package (Turner, 2014) was used to plot Manhattan and quantile-quantile (QQ) plots. A 5% significance level was used to identify SNPs significantly associated with anthracnose resistance. In the present study, we used linear scale for phenotypic measurements thus, smaller values represent resistance and larger values indicate susceptibility. Hence, SNPs with lower marker effect (**Supplementary Tables 3**-**7**) are linked to bean anthracnose resistance as per the procedure described by Mourad et al. (2018).

### Candidate Gene Identification

Significant SNPs for anthracnose resistance obtained from MLM analysis were blasted against the *P. vulgaris* v 2.0 available at www.phytozome.jgi.doe.gov for candidate gene identification. Gene annotation and sequence flanking (200 bp) for SNPs was also obtained from the phytozome. The top 10 SNPs were also checked at phytozome for functional annotation.

## Results

### Identification of Resistant Sources to Five Anthracnose Races

**Figure 2:**
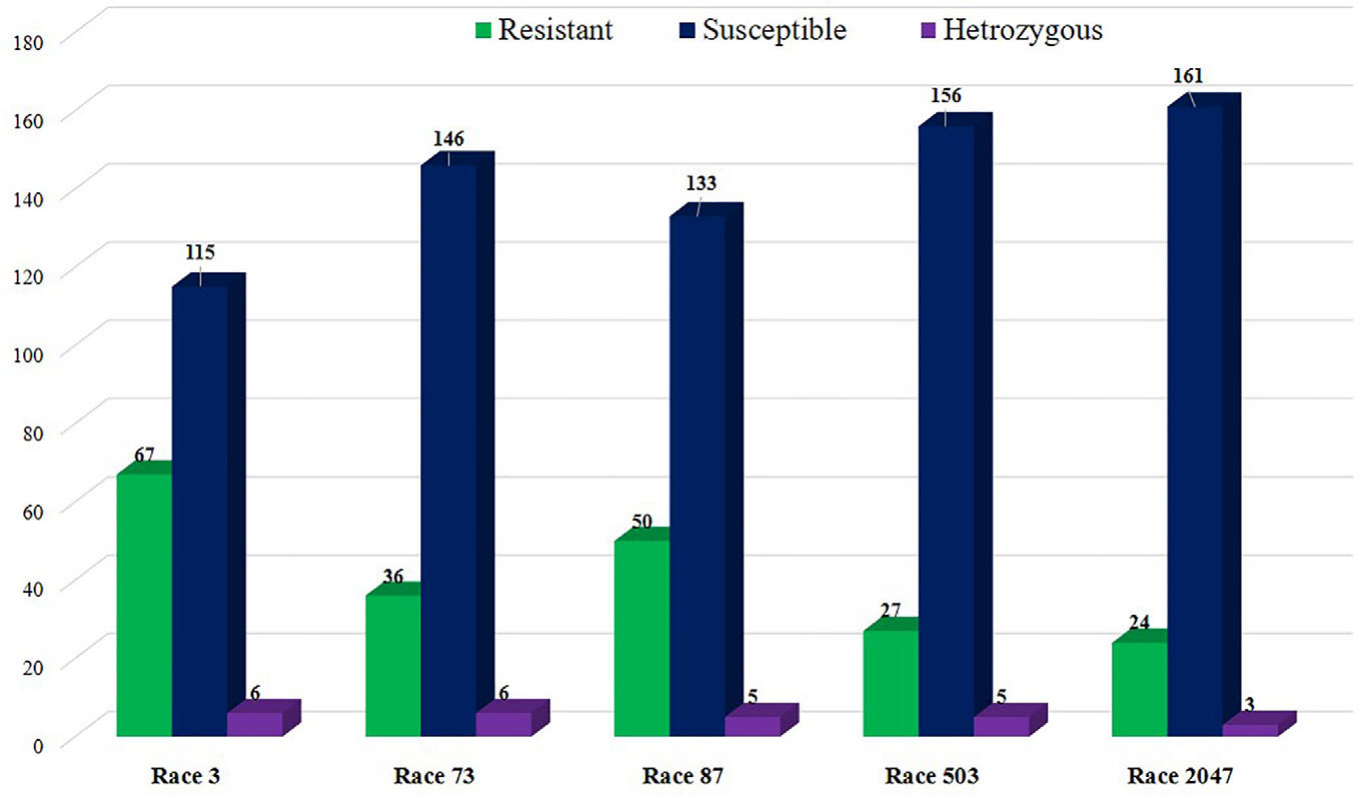
Reaction pattern of 188 north-western Himalayan land races to five bean anthracnose races.

Common bean genotypes (188) showed differential interaction when evaluated for anthracnose resistance with five anthracnose races. Genotypes that showed resistance against races were either immune or exhibited pinhead symptoms on leaves and stems after 3 days of inoculation (**Figure 1D**). Such cultivars showed hypersensitive response after 7 days of incubation. In contrary susceptible cultivars after 7 days showed severe infection on the cotyledons and the hypocotyls as small blackish sunken lesions that led to plant death (**Figure 1C**). Numerous anthracnose resistant lines were identified among the bean diversity panel. Out of 188 accessions, 113 were resistant to one or multiple races and 75 lines were susceptible to all races (**Supplementary Table 8** and **Figure 2**). Common bean genotype WB-1634 and WB-967 collected from NBPGR, Shimla showed resistance to all the five races, whereas WB-716 (NBPGR, Shimla) was resistant to four races. WB-1637 was resistant to races 2047, 3, 87, and 503. Bean anthracnose race 2047 was highly virulent and only 12.5% of the cultivars exhibited resistance to it, followed by race 503 for which 14.36% of the genotypes showed resistance. Anthracnose races 73 and 87 were unable to infect 18.7% and 26.0% cultivars, respectively. In contrary to other races, anthracnose race 3 was less virulent, as 34.8% of the bean genotypes were resistant to it (**Supplementary Table 8** and **Figure 2**). A number of anthracnose resistant bean genotypes were identified against the highly virulent anthracnose race 2047.

### Population Structure, Kinship Matrix, and Linkage Disequilibrium (LD)

**Figure 3:**
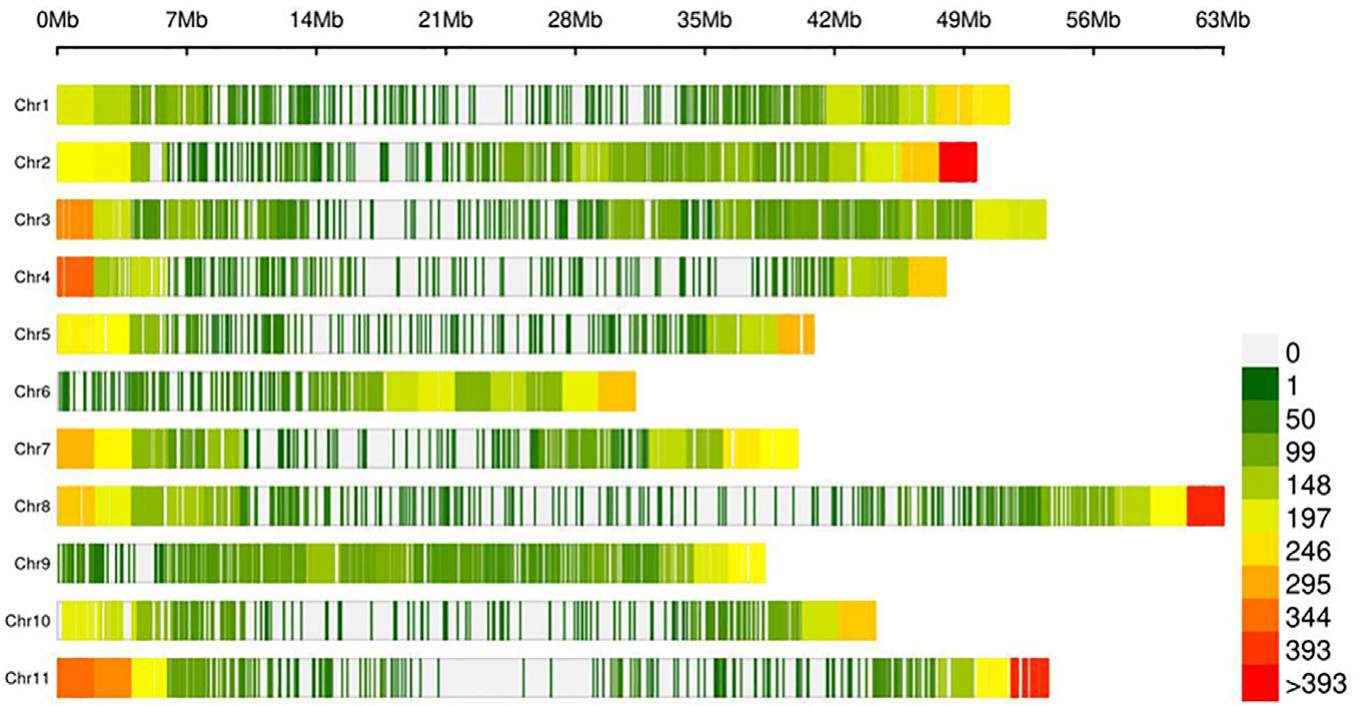
Density of 22,589 SNP within 1-Mb common bean genome window size. The different colors represent different density levels and “Chr” refers to common bean chromosomes.

**Figure 4:**
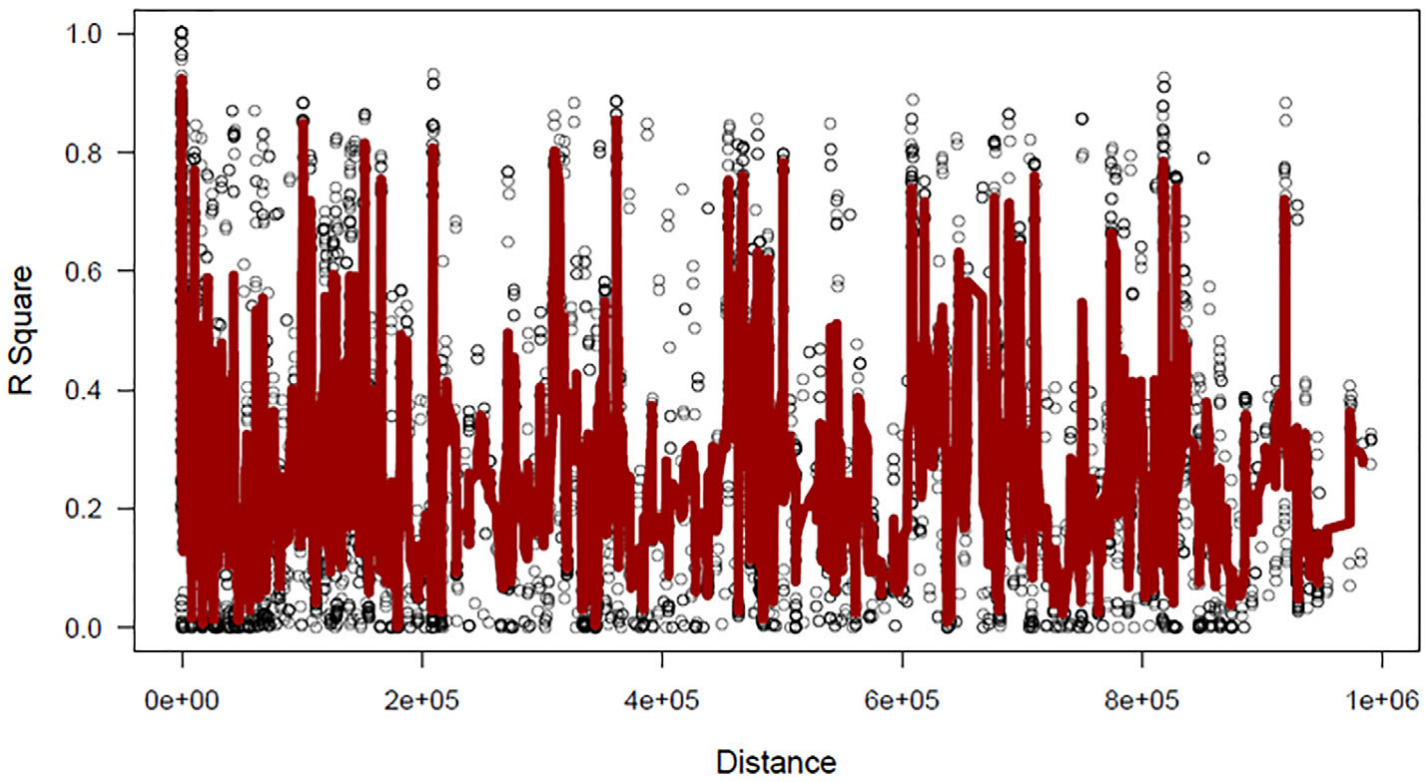
Linkage disequilibrium based on 22,589-filtered common bean SNP markers.

**Figure 5:**
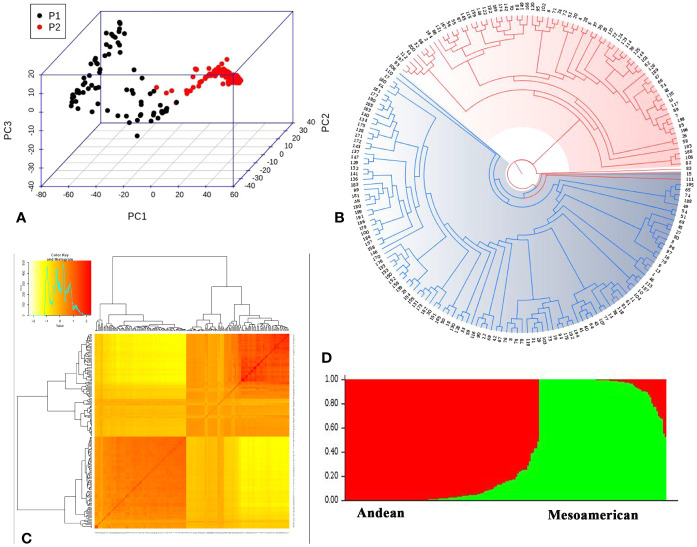
Population structure of 192 common beans inferred using different software’s. **(A)** Principal Component analysis performed using 22,598 evenly distributed SNPs along the bean genome. The two groups of accessions (P1 and P2) are depicted; one with black dots (Andean genotypes; P1) and dots with red color depict Mesoamerican genotypes (P2). **(B)** Phylogenetic tree generated using SNPhylo categorized 192 common bean genotypes into groups; Andean group (Blue) and Mesoamerican group (red). **(C)** Kinship matrix depicting pairwise similarities among 192 common bean genotypes; yellow and dark yellow color shows high similarities whereas red color depicts low similarity between the bean genotypes. **(D)** Genetic structure of 192 common bean genotypes based on model based approach implemented in STRUCTRE. Each genotypes is represented by a column divided into two colored segments (red and green) whose length indicates the proportions of the genome attributed to the two main groups (y axis); Andean (red) and Mesoamerican (green).

The lack of evidence about the common bean population structure and the gene pools to which the NW Himalayan beans belong to prompted us to undertake these studies. We used 192 bean genotypes (4 controls plus 188 Indian beans) for GBS and after SNP calling we obtained 250,526 SNPs (**Supplementary Figure 1**). The SNPs obtained varied from 18,198 to 28,005 on 11 bean pesudomolecules. In addition, 10,511 SNPs were present on various bean scaffolds (**Supplementary Figure 1**). After applying various quality-filtering parameters (MAF >= 0.05, MAC >= 10, Missing Data <= 50%) 22,589 SNPs were retained for downstream analysis and these SNPs were evenly distributed on 11 bean chromosomes (**Figure 3**). Linkage decay was observed after 1.0-Mb distance (**Figure 4**) that has a practical significance for identifying significant trait association even with a fewer number of markers. Population structure analysis of 192 bean genotypes (188 genotypes from India plus 4 controls) with STRUCTURE, GAPIT and SNPhylo categorized 188 Indian beans into two major clusters (**Figures 5A–D**) representing Andean and Mesoamerican gene pools. Among the 188 bean genotypes 114 (60.63%) belonged to Andean group and 60 (31.92%) belonged to Mesoamerican group. Both PCA and STRUCTURE analysis showed the presence of admixture within the 188 bean genotypes (**Figures 5A, D**). The two controls used in the present study helped us to categorize the NW beans into two distinct gene pools (5b). The first two principal components accounted for more than 40% of the variation. The kinship matrix also grouped NW Himalayan bean into two main groups belonging to Andean and Mesoamerican types (**Figure 5C**). However, subdivisions within the two main groups are evident and the genotypes showing admixture are located in the centre of the heat map (**Figure 5C**).

### Genome Wide Association Study

After removing the controls and heterozygous lines, 180 common bean genotypes were retained for a GWAS analysis. The filtered SNPs were evenly distributed across all the 11 bean chromosomes and 22,589 SNPs were used to find the marker trait associations through GWAS. Our data set showed the presence of structure, so the MLM model implemented in GAPIT was used to find significant SNPs associated with five anthracnose races. The expected and observed –log(10)p values followed X = Y distribution along the diagonal line until the observed *p* values diverted from the expected *p* values, which further strengthens that MLM model fits the data set.

**Figure 6:**
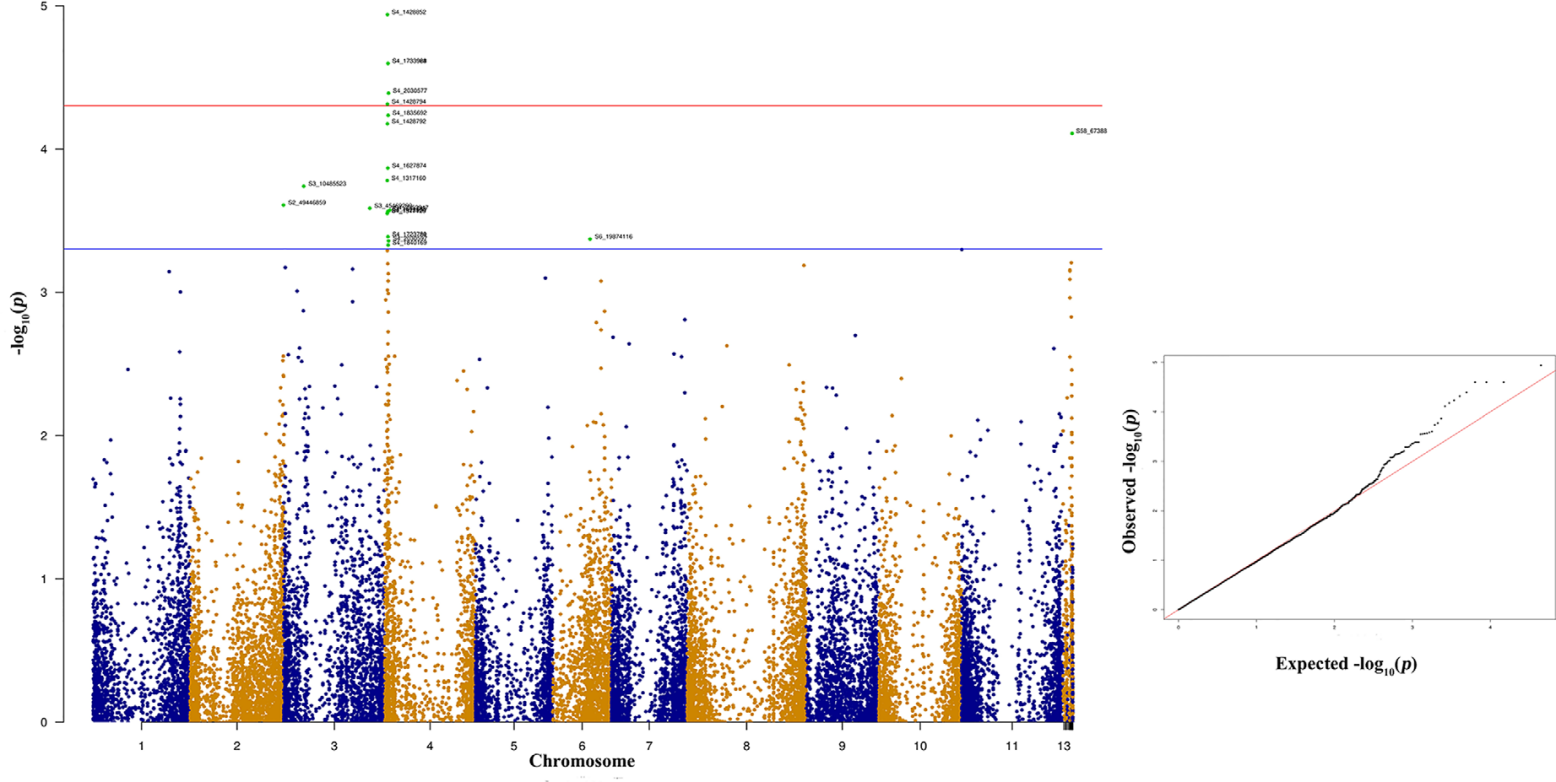
Manhattan and quantile-quantile (Q-Q) plots showing significant SNPs identified using MLM model that showed association with bean anthracnose race 3. Red line indicates Simple M Bonferroni corrected p-value threshold for significance and Green line indicates p-value of 1 × 10^3^.

The significant SNPs associated with anthracnose race 3 are listed in **supplementary Table 3** and the top 10 SNPs are listed in **Table 1**. There are many SNPs associated with anthracnose race 3 resistance at Pv04 (**Figure 6**) within the genomic region 1.31 to 2.03 Mb (**Table 1**). These SNPs contribute 16.71% to 19.57% of the phenotypic variation (**Table 1**). The other bean chromosome that contains a significant SNP associated with race 3 resistance is Pv03 and it contributes 16.62% of the phenotypic variation (**Table 1** and **Supplementary Table 3**).

**Table 1:** List of top 10 significant SNPs associated with anthracnose resistance against race 3.

SNP^#^	Chromosome	Position	p Value^$^	MAF^¥^	R^2^ (%)^€^	Allele effect
S4_1428852	*Pv04*	1428852	1.15E-05	0.42	19.57%	−1.09519
S4_1733922	*Pv04*	1733922	2.53E-05	0.45	18.72%	−1.16532
S4_1733964	*Pv04*	1733964	2.53E-05	0.45	18.72%	−1.16532
S4_1733985	*Pv04*	1733985	2.53E-05	0.45	18.72%	−1.16532
S4_2030577	*Pv04*	2030577	4.07E-05	0.23	18.20%	−1.1626
S4_1428794	*Pv04*	1428794	4.86E-05	0.27	18.01%	−0.98889
S4_1428792	*Pv04*	1428792	6.67E-05	0.27	17.67%	−0.97352
S4_1627874	*Pv04*	1627874	0.0001358	0.50	16.92%	−1.13859
S4_1317160	*Pv04*	1317160	0.000166	0.25	16.71%	−1.02688
S3_10485523	*Pv03*	10485523	0.0001821	0.24	16.61%	−1.2163

^#^SNP, Single Nucleotide Polymorphic Code.

^$^p Value, Significance Level.

^¥^MAF, Minor Allele Frequency.

^€^R^2^, Percent phenotypic variation explained by SNP.

**Figure 7:**
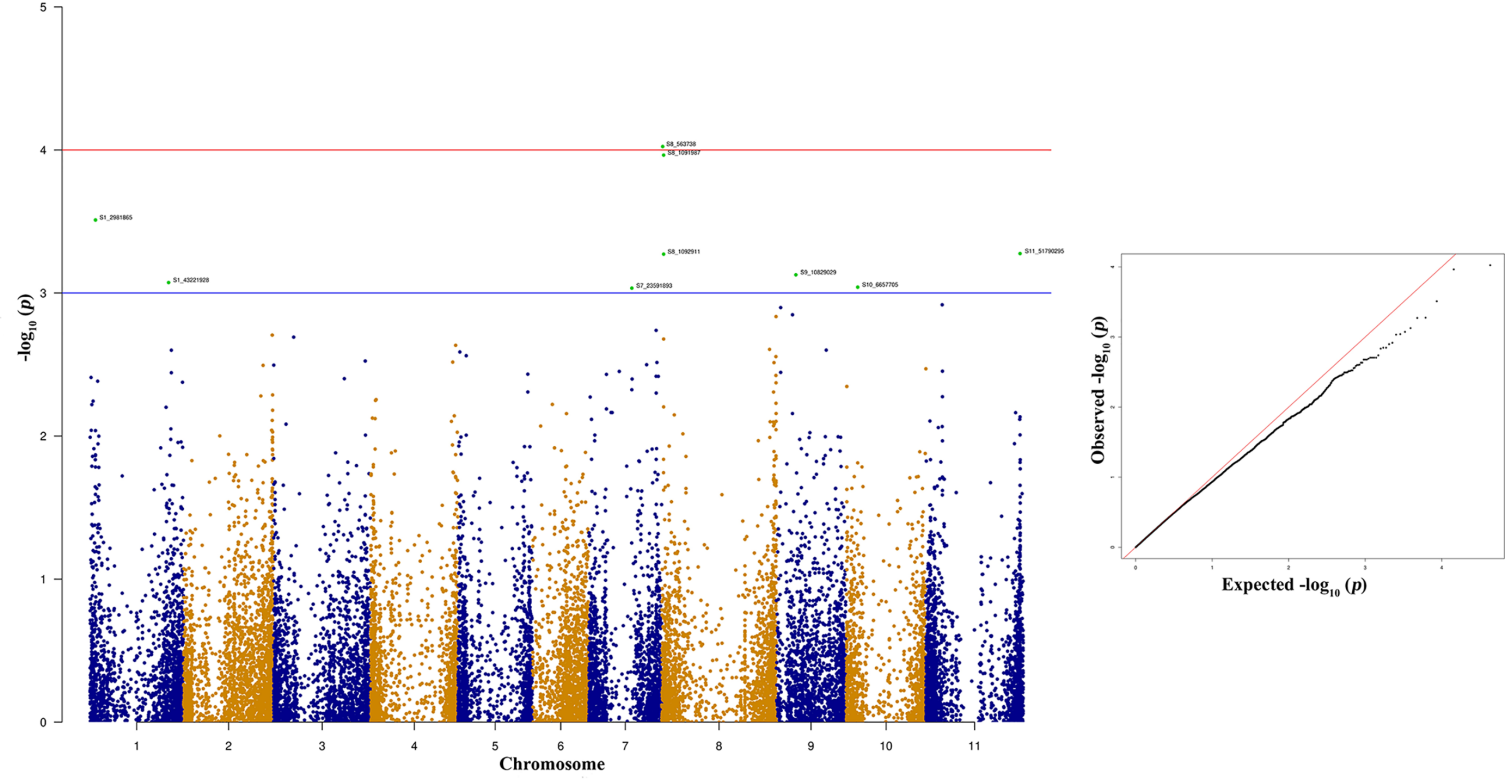
Manhattan and quantile-quantile (Q-Q) plots showing significant SNPs identified using MLM model that showed association with bean anthracnose race 73. Red line indicates Simple M Bonferroni corrected p-value threshold for significance and Green line indicates p-value of 1 × 10^3^.

The top two significant SNPs that showed association with race 73 resistance are located between genomic region 0.5 to 1.09 Mb at Pv08 (**Table 2**). There is one more SNP (S8_1092911) on Pv08 at 1.09-Mb physical position and it contributes 10.31% of the phenotypic variation. These SNPs cumulatively contribute about 35% phenotypic variation. The significant SNPs such as S8_563938 and S8_1091987 (**Figure 7** and **Supplementary Table 4**) indicate a major QTL associated with anthracnose race 73 resistance. The other significant SNPs among the top 10 SNPs associated with race 73 quantitative resistance are located at Pv01, Pv07, and Pv11 (**Figure 7** and **Table 2**).

**Table 2:** List of top 10 significant SNPs associated with anthracnose resistance against race 73.

SNP^#^	Chromosome	Position	p Value^$^	MAF^¥^	R^2^ (%)^€^	Allele effect
S8_563738	*Pv08*	563738	9.46E-05	0.17	12.23%	−1.07
S8_1091987	*Pv08*	1091987	0.0001086	0.16	12.08%	−1.14
S1_2981865	*Pv01*	2981865	0.0003088	0.17	10.91%	−1.20
S11_51790295	*Pv11*	51790295	0.0005302	0.15	10.32%	−1.08
S8_1092911	*Pv08*	1092911	0.0005349	0.16	10.31%	−0.99
S9_10829029	*Pv09*	10829029	0.0007462	0.40	9.95%	−1.80
S1_43221928	*Pv01*	43221928	0.0008447	0.49	9.82%	−0.81
S10_6657705	*Pv10*	6657705	0.0009103	0.30	9.73%	−0.90
S7_23591893	*Pv07*	23591893	0.0009247	0.16	9.72%	−0.84
S8_1091438	*Pv08*	1091438	0.0020972	0.16	8.84%	−0.88

^#^SNP, Single Nucleotide Polymorphic Code.

^$^p Value, Significance Level.

^¥^MAF, Minor Allele Frequency.

^€^R^2^, Percent phenotypic variation explained by SNP.

**Figure 8:**
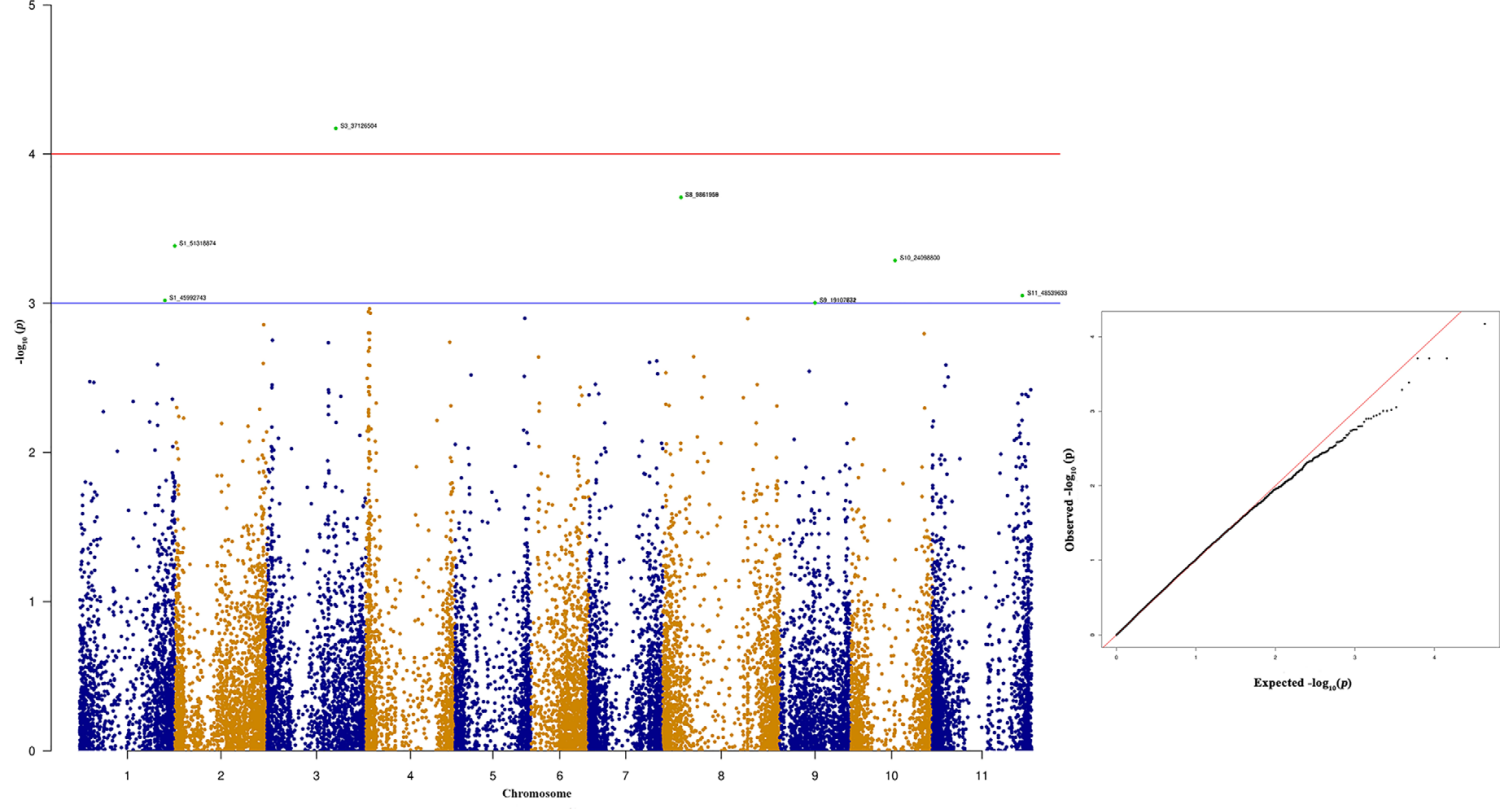
Manhattan and quantile-quantile (Q-Q) plots showing significant SNPs identified using MLM model that showed association with bean anthracnose race 87. Red line indicates Simple M Bonferroni corrected p-value threshold for significance and Green line indicates p-value of 1 × 10^3^.

Among the top 10 SNPs associated with race 87 resistance, six SNPs are located at Pv08 and Pv04 (**Figure 8** and **Table 3**). However, SNP, S3_37126504 located at Pv03, contributes 12.29% of the phenotypic variation. Three SNPs located at Pv08 explain 11.09% phenotypic variation. It was interesting to observe that the genomic region from 1.3 to 2.3 Mb at Pv04 is associated with race 87 and the region overlaps with genomic region associated with race 3. The distal end of Pv01 at 51.31 Mb contains another significant SNP that explains 10.27% phenotypic variation (**Table 3**). The distal end of Pv01 is known to contain many major anthracnose resistance genes and we hypnotize that this a most probable region for anthracnose resistance against race 87.

**Table 3:** List of top 10 significant SNPs associated with anthracnose resistance against race 87.

SNP^#^	Chromosome	Position	p Value^$^	MAF^¥^	R^2^ (%)^€^	Allelic effect
S3_37126504	Pv03	37126504	6.73E-05	0.22	12.29%	−1.42548
S8_9861950	Pv08	9861950	0.000195	0.43	11.09%	−1.47378
S8_9861956	Pv08	9861956	0.000195	0.43	11.09%	−1.47378
S8_9861959	Pv08	9861959	0.000195	0.43	11.09%	−1.47378
S1_51318874	Pv01	51318874	0.000412	0.41	10.27%	−1.25167
S11_48539633	Pv11	48539633	0.000887	0.43	9.43%	−1.28501
S4_1919854	Pv04	1919854	0.001088	0.21	9.21%	−0.85776
S4_1317160	Pv04	1317160	0.001141	0.25	9.16%	−0.78689
S4_2356775	Pv04	2356775	0.001164	0.26	9.14%	−1.09071
S5_37679523	Pv05	37679523	0.00126	0.42	9.05%	−1.45632

^#^SNP, Single Nucleotide Polymorphic Code.

^$^p Value, Significance Level.

^¥^MAF, Minor Allele Frequency.

^€^R^2^, Percent phenotypic variation explained by SNP.

**Figure 9:**
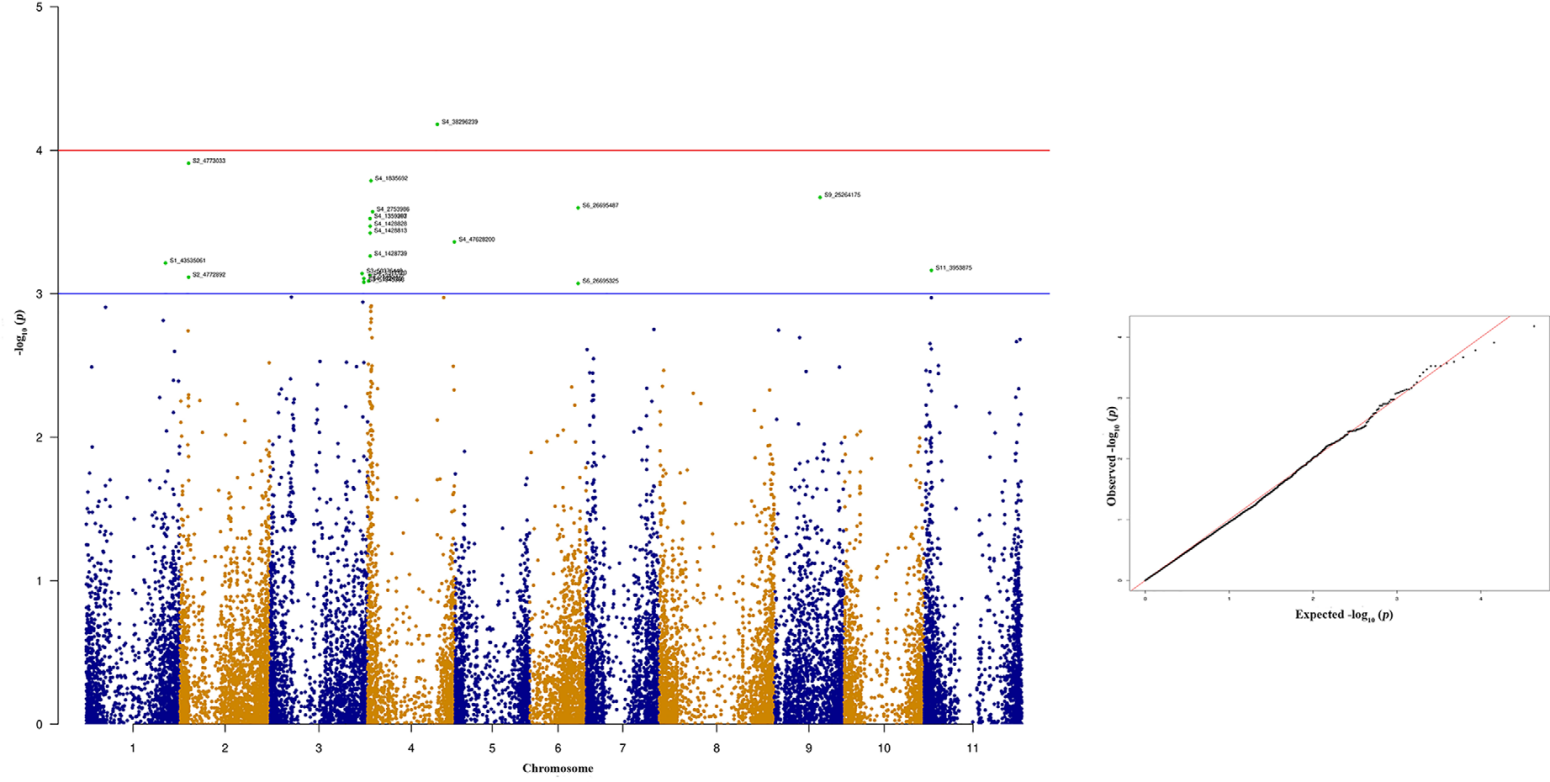
Manhattan and quantile-quantile (Q-Q) plots showing significant SNPs identified using MLM model that showed association with bean anthracnose race 503. Red line indicates Simple M Bonferroni corrected p-value threshold for significance and Green line indicates p-value of 1 × 10^3^.

Seven out of ten of the most significant SNPs that showed strong association with quantitative anthracnose resistance against race 503 are located at Pv04 which explains above 12.30% of the phenotypic variation (**Figure 9** and **Table 4**). Six of these SNPs are located between 1.35 to 2.72 Mb in the physical map of Pv04 and one is located at 47.62 Mb.

**Table 4:** List of top 10 significant SNPs associated with anthracnose resistance against race 513.

SNP^#^	Chromosome	Position	p Value^$^	MAF^¥^	R^2^ (%)^€^	Allele effect
S2_4773033	Pv02	4773033	0.000123	0.23	13.69%	−0.87535
S9_25264175	Pv09	25264175	0.000213	0.41	13.09%	−1.34983
S6_26695487	Pv06	26695487	0.000252	0.25	12.91%	−0.93768
S4_2753986	Pv04	2753986	0.000268	0.24	12.84%	−0.98211
S4_1359293	Pv04	1359293	0.000299	0.34	12.72%	−0.75623
S4_1359297	Pv04	1359297	0.000299	0.34	12.72%	−0.75623
S4_1359303	Pv04	1359303	0.000299	0.34	12.72%	−0.75623
S4_1428828	Pv04	1428828	0.000338	0.22	12.59%	−0.69919
S4_1428813	Pv04	1428813	0.000377	0.22	12.47%	−0.69725
S4_47628200	Pv04	47628200	0.000435	0.43	12.32%	−1.06486

^#^SNP, Single Nucleotide Polymorphic Code.

^$^p Value, Significance Level.

^¥^MAF, Minor Allele Frequency.

^€^R^2^, Percent phenotypic variation explained by SNP.

**Figure 10:**
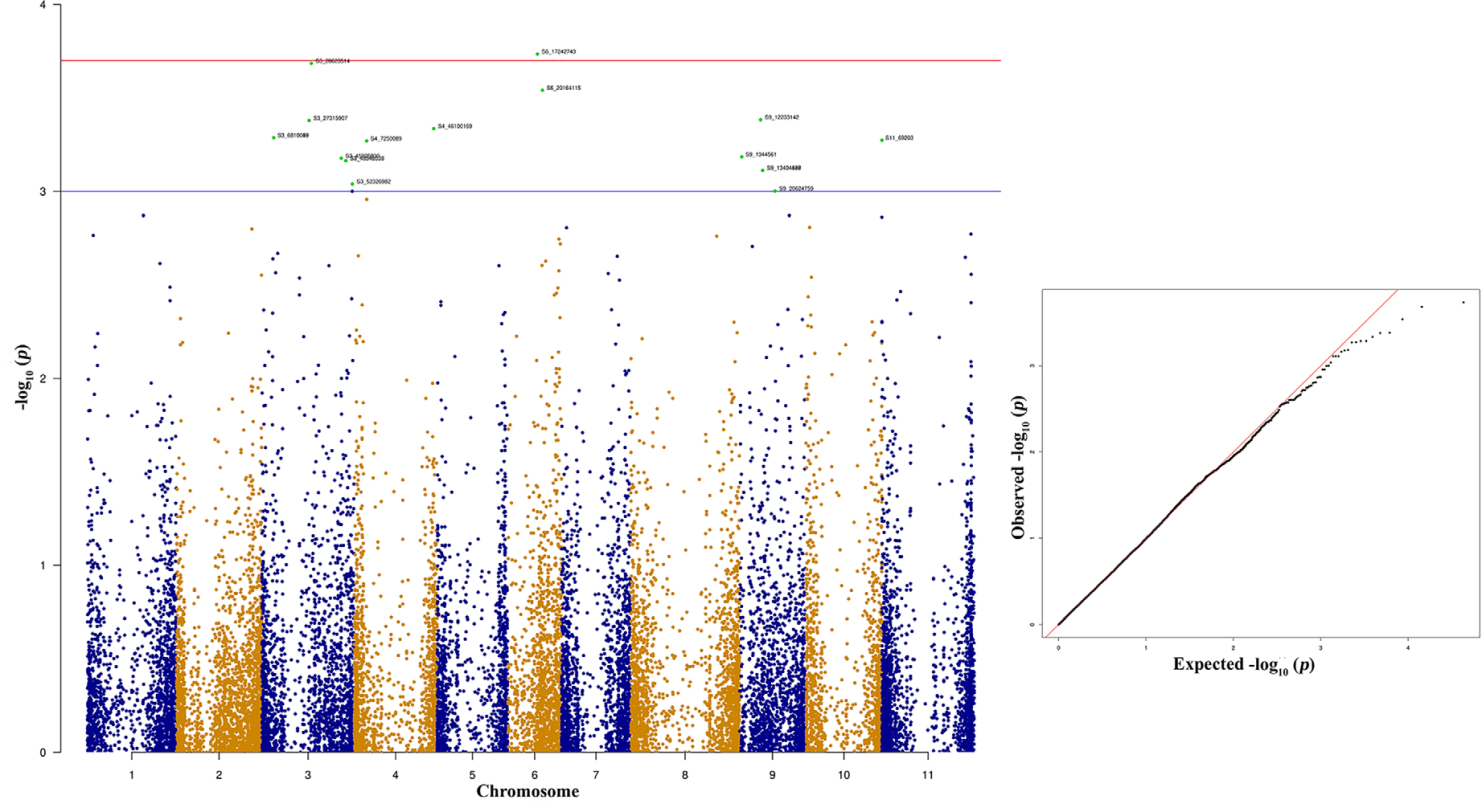
Manhattan and quantile-quantile (Q-Q) plots showing significant SNPs identified using MLM model that showed association with bean anthracnose race 2047. Red line indicates Simple M Bonferroni corrected p-value threshold for significance and Green line indicates p-value of 1 × 10^3^.

The top 10 significant SNPs associated with race 2047 resistance were distributed across the different bean chromosomes Pv03, Pv04, Pv06, and Pv09 (**Figure 10** and **Table 5**). These top 10 significant SNPs explain phenotypic variation between 6.98% and 8.47% (**Table 5**). Bean genomic regions at Pv03 with physical positions of 28.62 and 27.317 Mb are associated with race 2047 resistance. The other two SNPs located at 6.18 Mb on Pv03 contribute 7.43% of the phenotypic variation (**Table 5**).

**Table 5:** List of top 10 significant SNPs associated with anthracnose resistance against race 2047.

SNP^#^	Chromosome	Position	p Value^$^	MAF^¥^	R^2^ (%)^€^	Allelic effect
S3_28623514	Pv03	28623514	0.000207	0.18	8.47%	−1.05434
S6_20164115	Pv06	20164115	0.000288	0.42	8.09%	−1.7348
S3_27315907	Pv03	27315907	0.000418	0.42	7.67%	−1.2944
S4_46100169	Pv04	46100169	0.000461	0.17	7.56%	−0.94628
S3_6810049	Pv03	6810049	0.000516	0.40	7.43%	−1.48744
S3_6810056	Pv03	6810056	0.000516	0.40	7.43%	−1.48744
S11_69203	Pv11	69203	0.000533	0.36	7.39%	1.168018
S9_1344561	Pv09	1344561	0.000654	0.41	7.17%	−1.48853
S3_45925930	Pv03	45925930	0.000665	0.19	7.15%	−1.04988
S9_13404457	Pv09	13404457	0.000772	0.43	6.98%	−1.24493

^#^SNP, Single Nucleotide Polymorphic Code.

^$^p Value, Significance Level.

^¥^MAF, Minor Allele Frequency.

^€^R^2^, Percent phenotypic variation explained by SNP.

### Genomic Regions Associated With Anthracnose Resistance

It was interesting to observe that SNPs located at Pv04 are strongly associated with anthracnose resistance against races prevalent in NW Himalayan region. Many SNPs associated with race, 3, 87 and 503 are present up or downstream of genes having a strong role in disease resistance (**Supplementary Table 9**). For instance, common bean gene models Phvul.004G014800, Phvul.004G014801 and Phvul.004G015800 encode Leucine Rich Repeat (LRR) proteins. The common bean gene Phvul.004G015800 is having a typical NB-ARC domain (**Table 6**). A significant SNP (S9_13404457) located at Pv09 and associated with quantitative resistance against race 2047 encodes a serine threonine protein kinase (**Table 6**). In addition to the LRR region at Pv04 there is one more significant SNP (S9_25264175), which is located on Pv09 that encodes a zinc finger CCCH domain containing protein (**Table 6**). The other significant SNPs encode hypothetical proteins (**Supplementary Table 9**).

**Table 6:** Annotation of significant SNPs associated with different anthracnose resistance QTLs with typical role in disease resistance.

SNP^#^	Chromosome	Gene	Annotation
S4_1428852	Pv04	Phvul.004G012801	Lucien Rich Repeat
S4_1428813	Pv04	Phvul.004G012801	Lucien Rich Repeat
S4_1428828	Pv04	Phvul.004G012801	Lucien Rich Repeat
S4_1733922	Pv04	Phvul.004G015800	NBS-LRR with typical NB-ARC domain
S4_1733964	Pv04	Phvul.004G015800	NBS-LRR with typical NB-ARC domain
S4_1733985	Pv04	Phvul.004G015800	NBS-LRR with typical NB-ARC domain
S4_2753986	Pv04	Phvul.004G023900	Methyl transferase
S8_1091987	Pv08	Phvul.008G013300	Serine protease like
S8_1092911	Pv08	Phvul.008G013300	Serine protease like
S8_1091438	Pv08	Phvul.008G013300	Serine protease like
S9_25264175	Pv09	Phvul.009G169600	Zinc Finger CCCH Domain containing protein
S9_13404457	Pv09	Phvul.009G081200	Serine/Threonine Protein Kinase
S11_51790295	Pv11	Phvul.011G202300	Lucien Rich Repeat with typical NB-ARC domain
S7_23591893	Pv07	Phvul.007G144000	LysM4

## Discussion

### Sources of Anthracnose Resistance

*Phaseolus vulgaris – C. lindemuthianum* pathosystem generally follows gene for gene interaction and most of the identified resistance genes in bean genotypes are dominant except the *co-8* (Gonzalez et al., 2015). The pre-eminent approach to manage bean anthracnose is to identify broad spectrum resistant sources, exploit multitudinous genetic tools to unravel the underlying resistance mechanism in the resistant genotypes and then develop new resistant cultivars or deploy the identified cultivars spatially and temporally (Padder et al., 2010; Zuiderveen et al., 2016). In the present investigation, 188 bean genotypes from NW Himalayan region were screened against most prevalent five anthracnose races. Race 2047 is highly virulent (defeats all anthracnose resistance genes known in differentials except G2333) and only 4.4% genotypes from Andean diversity panel showed resistance to it (Zuiderveen et al., 2016). In NW region, 45 bean anthracnose races are reported and these are unable to infect G2333 and AB136 differential lines (Sharma et al., 2007; Sharma et al., 2019). Thus, it was anticipated that beans grown in northern India might show resistance against race 2047 because it is not reported in the bean growing areas but only 13.29% genotypes showed resistance, which is higher than the earlier reports (Zuiderveen et al., 2016). A good number of Andean beans such as WB967, WB-1634, WB-1637, and WB-716 showed resistance against multiple races and are good sources to start breeding programs for developing cultivars resistant to anthracnose race 2047. Similarly, we found a number of genotypes resistant to race 73, the most important race for the United States (Mahiya-Farooq et al., 2019). These genotypes can serve as a pool for breeding Andean and Mesoamerican genotypes. Anthracnose races 3, 87, and 503 are prevalent in the three northern states of India (Sharma et al., 2019) and the material resistant to these races can easily be exploited for the development of broad-spectrum anthracnose resistant cultivars for the region. The recent breakdown of the resistance gene present in AB136 by newly emerging races indicate coevolution of *C. lindemuthianum* in the region (Padder et al., 2009; Sharma et al., 2019) and warrants the effort to identify bean cultivars with resistance to multiple races. Although bean differential cultivars such as G2333, AB136 contain anthracnose resistance genes, their un-adaptability to NW Himalayan environment, hampers transfer of these genes to locally adapted cultivars. G2333 is photoperiod sensitive (Fernández et al., 2000; Ferreira et al., 2008; Goswami et al., 2011) similar to many anthracnose resistant genotypes identified by Fernández et al. (2000) and such cultivars are unsuitable for improvement of locally adapted beans. Alternatively, our material showed anthracnose resistance to race 2047 and resistance can easily be transferred to local commercially grown cultivars because these cultivars are adapted to the local environment and do not have limitations as those of G2333 and other differential cultivars. Over past few years, new anthracnose resistance genes were identified and mapped from diverse anthracnose resistant genotypes (Gonçalves-Vidigal et al., 2011; Richard et al., 2014; Lacanallo and Gonçalves-Vidigal, 2015; Trabanco et al., 2015; Chen et al., 2017; de Lima Castro et al., 2017). We expect our anthracnose resistant common bean material may contain diverse anthracnose resistance genes as the *C. lindemuthianum* virulence spectrum in NW region is different than the US, France and Canada (Padder et al., 2017).

### Population Structure

Based on STRUCTURE, SNPhylo, and Kinship matrix, our common bean material (188 lines) along with the four control genotypes (each from Andean and Mesoamerican gene pools) helped us to categorize NW common bean populations into two distinct populations. This is the comprehensive study that shows common beans grown in India belong to two gene pools. Previously, Sharma et al. (2013) genotyped 67 common bean landraces of Himachal Pradesh with SSR markers and showed existence of two distinct gene pools in the state. It is well documented that common beans are native to Andean and Mesoamerican regions (Gepts and Debouck, 1991; McConnell et al., 2010; Bitocchi et al., 2012) and our study shows existence of two distinct gene pools in India that indicates probably multiple introductions of common bean occurred from their native places in to the region. We also observed admixture in our material similar to European common beans. Sharma et al. (2013) hypnotized that common beans grown in the NW region may have introduced either eastward or westward. This indicates that the common beans in India might be introduced from Europe (via Africa) or China and is strongly supported by earlier studies (Joshi and Thomas, 1987; Joshi and Mehra, 1993; Sharma et al., 2013). However, comprehensive studies similar to Bitocchi et al. (2013) are needed to answer single or multiple introductions into the region.

### Association Mapping

A powerful tool currently available to scientific communities for QTL dissection is GWAS that provide higher resolution than QTL mapping (Muhammad et al., 2020). Hence, in recent years, use of GWAS for mapping economically important QTLs in agricultural crops has increased and *P. vulgaris* is not behind. Many QTLs having economic importance in common bean have been mapped using a GWAS approach (Moghaddam et al., 2016; Wu et al., 2017; Kelly and Bornowski, 2018; Kamfwa et al., 2019; Bisneta and Gonçalves-Vidigal, 2020; Bornowski et al., 2020; Gonçalves-Vidigal et al., 2020; Mungalu et al., 2020). In the present study, significant differences observed in NW Indian bean genotypes led to the identification of SNPs associated with the anthracnose resistance. Our findings resulted in the identification of new genomic regions associated with anthracnose resistance and to the best of our knowledge, it is the first study from India. Further, most of the GWAS studies explored bean chip to map economic traits in beans, however, present study explored GBS to find associations of SNPs with anthracnose resistance.

Many significant SNPs associated with anthracnose resistance to Indian *C. lindemuthianum* virulent races (3, 87, and 503) are located on Pv04. Most of these SNPs are either located up or down stream of gene models that encode LRR and have typical NB-ARC domains. Most of the plant disease resistance genes belong to the NBS-LRR class and play a significant roles in plant disease resistance (Jones and Dangl, 2006; Padder, 2014). The common bean genome also contains NBS-LRR regions that are distributed across all chromosomes (Ferrier-Cana et al., 2003; Richard et al., 2017; Wu et al., 2017) and a recent GWAS study showed associations between these NBS-LRR regions with anthracnose resistance (Wu et al., 2017). Anthracnose resistance genes Co-3 and its alleles, *Co-16* are mapped to common bean Pv04 and this study also found association of SNPs on Pv04 with anthracnose resistance to races 3, 87, and 503 (Geffroy et al., 1999; David et al., 2008; Coimbra-Gonçalves et al., 2016; Murube et al., 2019). The Co-3^4^ locus was previously mapped on the 3.36-Mb genomic position but it was recently mapped to the region between 0.49 to 0.58 Mb on Pv04 (Valentini et al., 2017) which indicates shift in the physical positions with better mapping tools (Kelly and Bornowski, 2018). A previous GWAS study with an Andean diversity panel by Zuiderveen et al. (2016) identified two SNPs located at 0.45 and 0.53 Mb on Pv04 against race 7 and 109, respectively. A recent study identified 21 kinases and 55 NBS-LRR encoding genes close to the major genes located on Pv04 (Bisneta and Gonçalves-Vidigal, 2020). In the current study, SNPs that showed strong association with race 3 and 503 were located between the physical positions of 1.3 to 2.75 Mb, which are close to the anthracnose resistance genes (Co-3 locus and *Co-16*) present on the proximal end of Pv04. We also found a gene model (Phvul.004G023900) that encodes a methyltransferase that impart quantitative resistance to race 503. The role of methyltransferases in plant disease resistance is well documented against an array of plant pathogens (Yang et al., 2017; Wang et al., 2018; Bulman et al., 2019). Besides Pv04, this study identified a SNP on Pv09 for quantitative resistance against race 503 in gene model Phvul.009G169600 that encodes a zinc finger protein. This gene model contains a CCCH domain and its involvement in plant disease resistance is well documented (Deng et al., 2012; Gao W. et al., 2016; Cui et al., 2018). In the common bean genome only two NBS-LRR genes are located at distal end of Pv09 (Richard et al., 2017) and identification of QTL against race 503 on Pv09 is interesting and warrants *in silico* identification of other plant disease resistance signatures such as zinc finger proteins from the common bean genomes.

The Co-1 locus is present on the distal end of Pv01 and contains five alleles that have been extensity used in breeding anthracnose resistant cultivars in the United States and Mexico (Melotto and Kelly, 2000; Goncalves-Vidigal and Kelly, 2006; Gonçalves-Vidigal et al., 2011). The *Co-1* gene is present at a physical position of 50.30 Mb and we recently found high expression of gene model Phvul.001G243800 that encodes receptor like kinase following inoculation of a resistant NIL with race 73 (Zuiderveen et al., 2016; Mahiya-Farooq et al., 2019). Interestingly we were unable to find any significant SNPs among the list of top 10 SNPs associated with anthracnose resistance against race 73. However, this study identified a SNP associated with race 503 quantitative resistance. This SNP is located at 51.31 Mb and is close to the Co-1 locus and we hypothesize the involvement of Co-1 locus with race 503.

In this study four out of top 10 significant SNPs associated with race 73 quantitate resistance are located on Pv08. These SNPs are located between 0.56- and 1.09-Mb physical positions and encode hypothetical proteins. The role of hypothetical proteins cannot be completely ruled out. There is a single major anthracnose resistance gene (*Co-4* and its alleles) present on Pv08 and a recent study fine mapped *Co-4* between physical position of 0.49 to 2.84 Mb (Burt et al., 2015). Hence, it can be concluded that the SNPs overlap with the *Co-4* anthracnose resistance gene. Identification of the *Co-4* gene in the NW Himalayan bean lines is of paramount importance because this gene is highly effective against numerous anthracnose races (Melotto and Kelly, 2001; Oblessuc et al., 2015). Another source of *Co-4*, G2333 is photosensitive and does not flower under North Indian conditions, therefore hampering its usefulness as a source for introgression of *Co-4* gene to Indian beans. The indication of presence of the *Co-4* gene in our material is encouraging and breeders can easily introgress this gene into elite material for breeding anthracnose resistant cultivars. Additionally, we found a SNP (S11_51790295) located at distal end of Pv11 (51.79 Mb) in a gene model Phvul.011G202300 that encodes a LRR with typical NB-ARC domain. This region contains many NBS-LRR genes (Richard et al., 2017). The *Co-2* gene was previously mapped at 39.73 Mb (Creusot et al., 1999; Zuiderveen et al., 2016), but a recent study fine mapped it to 51.52 Mb (Kelly and Bornowski, 2018; Miklas et al., 2020). There is a tight association between *Co-2* and *Ur-11*, a bean rust resistance gene (Awale et al., 2008). The present study identified a SNP that overlaps with the *Co-2* anthracnose resistance gene.

Different genomic regions are associated with quantitative resistance against race 2047. These regions belong to Pv03, Pv09, and Pv11 and the SNPs contribute less than 8.0% of the phenotypic variation. This may be because of the high virulence of race 2047 that defeats the entire major anthracnose resistance genes, except the three genes that are present in the differential cultivar, G2333. No significant SNP was found associated with anthracnose resistance against race 2047 in the Andean diversity panel (Zuiderveen et al., 2016) and the study also failed to identify any significant loci that was attributed to low level of resistance against it. The present study identified a significant SNP that is located on Pv11 and the gene model Phvul.009G081200 encodes a serine threonine protein kinase. Serine threonine protein kinases play significant role in plant disease resistance in many host pathogen interactions (Afzal et al., 2008; Juliana et al., 2018). There are a few effective anthracnose resistance genes against race 2047 such as *Co-4*, *Co-12*, *Co-13*, *Co-14*, and *Co-15* (Goncalves-Vidigal et al., 2009; Goncalves-Vidigal et al., 2012; Sousa et al., 2015). Two of our significant SNPs are located on Pv03 at 27.31 and 28.62 Mb and both encode hypothetical proteins. The other genes present on Pv03 have not been fine mapped and we assume that the SNPs associated with quantitate resistance against race 2047 are different from *Co-13* and *Co-17* anthracnose resistance genes. The *Co-17* gene was mapped to the extreme proximal end of Pv03 (Trabanco et al., 2015; Kelly and Bornowski, 2018; Bisneta and Gonçalves-Vidigal, 2020). Bisneta and Gonçalves-Vidigal (2020) found six genomic regions on Pv03 associated with bean anthracnose which are distant from the significant SNPs found associated with anthracnose resistance in the present study.

## Conclusion

The present study revealed many sources of resistance to different anthracnose races prevalent in Northern India and to the two important anthracnose races 73 and 2047. A few genotypes are resistant to all the races among them, including a red colored Andean bean WB967, which will be a good source of resistance to develop durable anthracnose resistant cultivars. The present study showed that beans grown in the NW Himalayan region belong to Andean and Mesoamerican gene pools. Many novel QTLs particularly at Pv03, Pv08, Pv09, and Pv11 were identified in the gene models that encode typical plant disease resistance proteins. These novel genomic regions may contain major anthracnose resistance genes that needs to be elucidated using genetic studies. We found many SNPs that overlap with *Co-4* anthracnose resistance gene and its presence in our material is encouraging for breeding durable anthracnose resistant cultivars for the region.

## Data Availability Statement

The original contributions presented in the study are included in the article/**Supplementary Material**; further inquiries can be directed to the corresponding author.

## Author Contributions

AB, AN, and RR multiplied common bean genotypes. M-F and A-N maintained the anthracnose race cultures. Phenotyping of bean genotypes was performed by AB, AN, RR, A-N, and MS. PAS and PNS provided the material 192 common bean genotypes. MA and HI helped in multiplication of genotypes at their respective stations. The whole work was supervised by BP. BP designed the study, analyzed SNP data set, and wrote/edited the MS. All authors contributed to the article and approved the submitted version.

## Conflict of Interest

The authors declare that the research was conducted in the absence of any commercial or financial relationships that could be construed as a potential conflict of interest.

The reviewer JK declared a past co-authorship with one of the authors BP to the handling editor.
