# Supplementary figures and images for: North-Western Himalayan Common Beans: Population Structure and Mapping of Quantitative Anthracnose Resistance Through Genome Wide Association Study
Source: Front Plant Sci. 2020 Oct 6;11:571618. doi: 10.3389/fpls.2020.571618 (PMC7573075; doi:10.3389/fpls.2020.571618)

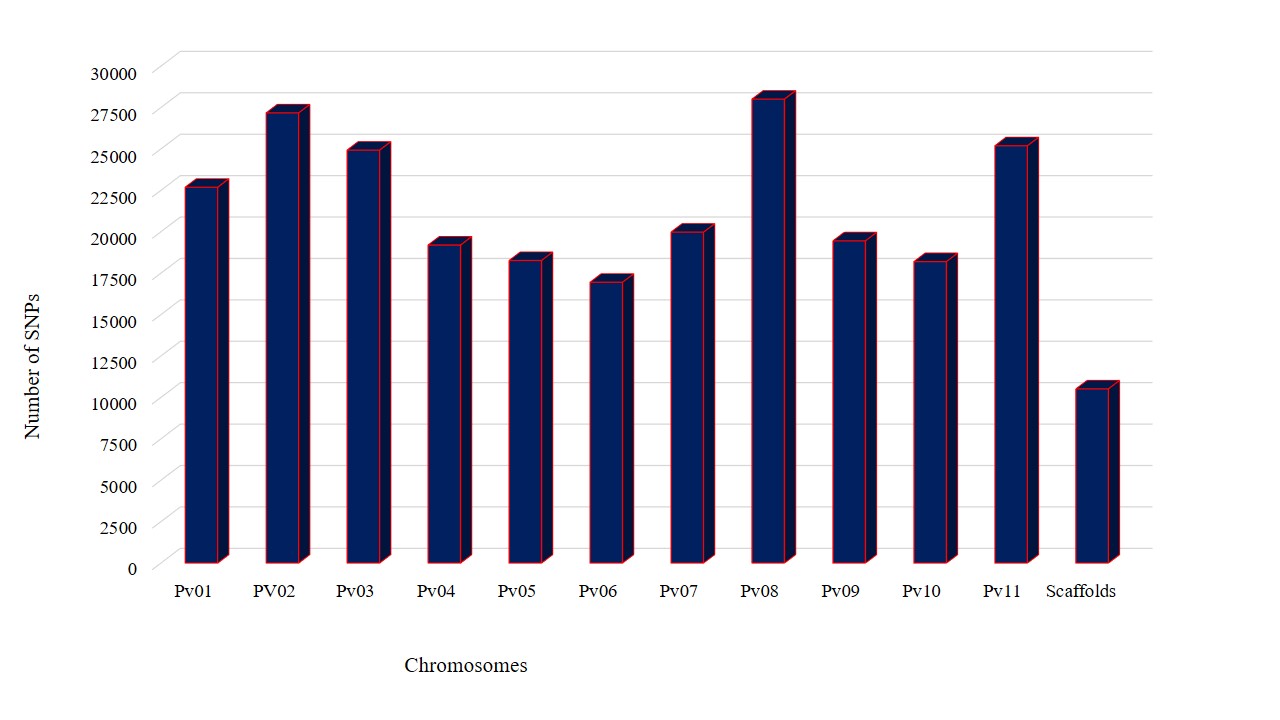

Supplement: Supplementary file 1 [file Image_1.jpeg]
