# Supplementary material for: North-Western Himalayan Common Beans: Population Structure and Mapping of Quantitative Anthracnose Resistance Through Genome Wide Association Study
Source: Front Plant Sci. 2020 Oct 6;11:571618. doi: 10.3389/fpls.2020.571618 (PMC7573075; doi:10.3389/fpls.2020.571618)
Supplement: Supplementary file 2 [file Table_1.docx]

Supplementary Table 1. Passport data of 192 bean genotypes that were used in the present study

| S. No. | Genomic DNA ID | Genotype | Location |
| --- | --- | --- | --- |
| 1 | 1 | WB-1255 | Local |
| 2 | 2 | WB-1318 | Local |
| 3 | 3 | WB-1184 | Local |
| 4 | 4 | WB-836 | Local |
| 5 | 5 | WB-206 | FOA, Wadura |
| 6 | 6 | WB-1316 | Local |
| 7 | 7 | WB-1492 | NBPGR, Shimla |
| 8 | 8 | WB-437 | NBPGR, Shimla |
| 9 | 9 | WB-1181 | Local |
| 10 | 10 | WB-1436 | NBPGR, Shimla |
| 11 | 11 | WB-1274 | Local |
| 12 | 12 | WB-1446 | NBPGR, Shimla |
| 13 | 13 | WB-1182 | Local |
| 14 | 14 | WB-1190 | Local |
| 15 | 15 | WB-1151 | Local |
| 16 | 16 | WB-1697 | Local |
| 17 | 17 | KRC-8 | CSK HPKV, Palampur |
| 18 | 18 | WB-1171 | Local |
| 19 | 19 | WB-1304 | Local |
| 20 | 20 | WB-1319 | Local |
| 21 | 22 | WB-1137 | Local |
| 22 | 23 | WB-1136 | Local |
| 23 | 24 | WB-1664 | Local |
| 24 | 25 | WB-1634 | NBPGR, Shimla |
| 25 | 26 | WB-1150 | Local |
| 26 | 27 | WB-1643 | NBPGR, Shimla |
| 27 | 28 | WB-1643 | NBPGR, Shimla |
| 28 | 29 | WB-1131 | Local |
| 29 | 30 | WB-1677 | Local |
| 30 | 31 | WB-1129 | Local |
| 31 | 32 | WB-893 | Local |
| 32 | 33 | WB-1177 | Local |
| 33 | 34 | IC-313295 | NBPGR, Shimla |
| 34 | 35 | 22721 | NBPGR, Shimla |
| 35 | 37 | IC-285575 | NBPGR, Shimla |
| 36 | 38 | 772 | Local |
| 37 | 39 | G-22552 | NBPGR, Shimla |
| 38 | 40 | Jawala | CSK HPKV, Palampur |
| 39 | 41 | WB-4564 | NBPGR, Shimla |
| 40 | 42 | WB-5176 | Local |
| 41 | 43 | WB-6960 | NBPGR, Shimla |
| 42 | 44 | WB-1282 | Local |
| 43 | 45 | WB-1185 | Local |
| 44 | 46 | IC-132 | Local |
| 45 | 47 | WB-901 | Local |
| 46 | 48 | WB-869 | Local |
| 47 | 49 | WB-864 | Local |
| 48 | 50 | WB-846 | Local |
| 49 | 51 | WB-832 | Local |
| 50 | 53 | WB-489 | Local |
| 51 | 54 | WB-482 | Local |
| 52 | 55 | WB-451 | NBPGR, Shimla |
| 53 | 56 | WB-401 | NBPGR, Shimla |
| 54 | 58 | WB-920 | Local |
| 55 | 59 | WB-923 | Local |
| 56 | 61 | SR-1 |  |
| 57 | 62 | D-line | CSK HPKV, Palampur |
| 58 | 64 | KRC-5 | CSK HPKV, Palampur |
| 59 | 65 | IC-313295 | NBPGR, Shimla |
| 60 | 66 | WB-1691 | Local |
| 61 | 67 | WB-956 | NBPGR, Shimla |
| 62 | 68 | WB-1006 | Local |
| 63 | 69 | WB-967 | NBPGR, Shimla |
| 64 | 71 | WB-507 | Local |
| 65 | 72 | WB-1438 | NBPGR, Shimla |
| 66 | 73 | WB-630 | Local |
| 67 | 74 | WB-642 | Local |
| 68 | 75 | WB-634 | Local |
| 69 | 76 | WB-650 | Local |
| 70 | 77 | WB-643 | Local |
| 71 | 78 | WB-665 | Local |
| 72 | 79 | WB-651 | Local |
| 73 | 80 | WB-662 | Local |
| 74 | 81 | WB-716 | NBPGR, Shimla |
| 75 | 82 | WB-371 | NBPGR, Shimla |
| 76 | 83 | WB-352 | NBPGR, Shimla |
| 77 | 84 | WB-335 | NBPGR, Shimla |
| 78 | 85 | WB-333 | Local |
| 79 | 86 | WB-258 | Local |
| 80 | 87 | WB-252 | Local |
| 81 | 89 | WB-115 | Local |
| 82 | 90 | WB-243 | Local |
| 83 | 93 | WB-242 | Local |
| 84 | 94 | WB-216 | FOA, Wadura |
| 85 | 95 | WB-195 | FOA, Wadura |
| 86 | 96 | WB-185 | NBPGR, Shimla |
| 87 | 97 | WB-191 | FOA, Wadura |
| 88 | 98 | WB-186 | Local |
| 89 | 99 | WB-112 | Local |
| 90 | 100 | PBG-102 | Local |
| 91 | 101 | WB-92 | NBPGR, Shimla |
| 92 | 102 | WB-83 | Local |
| 93 | 103 | WB-46 | Local |
| 94 | 104 | WB-22 | NBPGR, Shimla |
| 95 | 106 | WB-5178 | Local |
| 96 | 107 | WB-1587 | NBPGR, Shimla |
| 97 | 108 | WB-1690 | Local |
| 98 | 110 | WB-1310 | Local |
| 99 | 111 | GLY-P | Local |
| 100 | 112 | WB-1490 | NBPGR, Shimla |
| 101 | 113 | PBG-545 | Local |
| 102 | 114 | WB-765 | Local |
| 103 | 115 | 24044C | NBPGR, Shimla |
| 104 | 116 | WB-373 | NBPGR, Shimla |
| 105 | 117 | WB-916 | Local |
| 106 | 118 | WB-957 | NBPGR, Shimla |
| 107 | 119 | 10928 | Local |
| 108 | 120 | WB-1710 | Local |
| 109 | 121 | WB-1670 | Local |
| 110 | 122 | WB-1518 | NBPGR, Shimla |
| 111 | 123 | WB-45 | Local |
| 112 | 124 | WB-1703 | Local |
| 113 | 125 | WB-953 | NBPGR, Shimla |
| 114 | 126 | IC-321181 | NBPGR, Shimla |
| 115 | 127 | IC-530923 | NBPGR, Shimla |
| 116 | 128 | WB-487 | NBPGR, Shimla |
| 117 | 129 | IC-4850 | NBPGR, Shimla |
| 118 | 130 | WB-1699 | Local |
| 119 | 131 | WB-1698 | Local |
| 120 | 132 | WB-1682 | Local |
| 121 | 133 | WB-4709 | NBPGR, Shimla |
| 122 | 134 | WB-218 | FOA, Wadura |
| 123 | 135 | IC-319825 | NBPGR, Shimla |
| 124 | 136 | WB-1256 | Local |
| 125 | 137 | WB-565 | NBPGR, Shimla |
| 126 | 138 | WB-1702 | Local |
| 127 | 139 | WB-1685 | Local |
| 128 | 140 | WB-228 | FOA, Wadura |
| 129 | 141 | WB-1704 | Local |
| 130 | 142 | PBG-58 | Local |
| 131 | 143 | WB-955 | NBPGR, Shimla |
| 132 | 144 | WB-1680 | NBPGR, Shimla |
| 133 | 145 | WB-934 | NBPGR, Shimla |
| 134 | 146 | WB-429 | CSK HPKV, Palampur |
| 135 | 147 | R-121 | Local |
| 136 | 148 | WB-1139 | Local |
| 137 | 149 | IC-21529 | NBPGR, Shimla |
| 138 | 150 | WB-1496 | NBPGR, Shimla |
| 139 | 151 | PM-1 | Local |
| 140 | 152 | WB-1678 | Local |
| 141 | 153 | WB-6675 | NBPGR, Shimla |
| 142 | 154 | WB-1249 | Local |
| 143 | 155 | WB-1455 | NBPGR, Shimla |
| 144 | 156 | WB-255 | Local |
| 145 | 157 | WB-418 | NBPGR, Shimla |
| 146 | 158 | WB-1707 | Local |
| 147 | 159 | SSGB-729 | Local |
| 148 | 160 | SKR-925 | Local |
| 149 | 161 | DARS-38 | Local |
| 150 | 162 | DARS-54 | Local |
| 151 | 163 | DARS-17 | Local |
| 152 | 164 | French Yellow | Local |
| 153 | 165 | KDFB-81 | Local |
| 154 | 166 | KDR-97 | Local |
| 155 | 167 | ICB-002 | Local |
| 156 | 168 | SKR-23 | Local |
| 157 | 169 | KBR-63 | Local |
| 158 | 170 | WB-1709 | Local |
| 159 | 171 | Arka Anup | Local |
| 160 | 172 | WB-1705 | Local |
| 161 | 173 | WB-1693 | Local |
| 162 | 174 | KDR-98 | Local |
| 163 | 175 | DARS-10-1 | Local |
| 164 | 176 | Local Tral-2 | Local |
| 165 | 177 | KDFD-7 | Local |
| 166 | 178 | SKR-91 | Local |
| 167 | 179 | SKR-204 | Local |
| 168 | 180 | SKR-928 | Local |
| 169 | 181 | DARS-40 | Local |
| 170 | 182 | DARS-9 | Local |
| 171 | 183 | Local Tral-1 | Local |
| 172 | 184 | N-4 | Local |
| 173 | 185 | DARS-43 | Local |
| 174 | 186 | DARS-16 | Local |
| 175 | 187 | DARS-109 | Local |
| 176 | 188 | KDFD-3 | Local |
| 177 | 189 | DARS-39 | Local |
| 178 | 190 | DARS-11 | Local |
| 179 | 191 | Madwan | Local |
| 180 | 192 | Selection-3 | Local |
| 181 | 193 | KDR-96 | Local |
| 182 | 194 | DARS-10 | Local |
| 183 | 195 | R-132 | Local |
| 184 | 196 | GLC-P | Local |
| 185 | 197 | GLS-P | Local |
| 186 | 198 | GLM-P | Local |
| 187 | 199 | GLS-L | Local |
| 188 | 200 | WB-1630 | NBPGR, Shimla |
| 189 | 201 | AN | CSK HPKV, Palampur |
| 190 | 202 | KABOON | CSK HPKV, Palampur |
| 191 | 203 | MICHILITE | CSK HPKV, Palampur |
| 192 | 204 | TO | CSK HPKV, Palampur |

Supplementary Table 2. Disease reaction of bean anthracnose differential set to five *Colletotrichum lindemuthianum* races

| Race | Differential set^#^ | | | | | | | | | | | |
| --- | --- | --- | --- | --- | --- | --- | --- | --- | --- | --- | --- | --- |
|  | A | B | C | D | E | F | G | H | I | J | K | L |
| 3 | + | + | - | - | - | - | - | - | - | - | - | - |
| 73 | + | - | + | - | - | + | - | - | - | - | - | - |
| 87 | + | + | + | - | + | - | + | - | - | - | - | - |
| 503 | + | - | - | + | + | + | + | + | + | - | - | - |
| 2047 | + | + | + | + | + | + | + | + | + | + | + | - |

+ = susceptible; − = resistant;

#: Differential cultivars and their binary values (In parentheses): A-Michelite (1); B- Michigan dark red kidney(2); C- Perry Marrow (4); D Cornell 49,242 (8); E- Widusa (16); F- Kaboon (32); G-Mexique 222 (64); H- PI 207262(128); I-TO (256); J-TU (512); K-AB136 (1024); and L-G2333(2048)

Supplementary Table 8: List of 113 NW bean genotypes with resistance to one or multiple anthracnose race(s)

| **S. No.** | **Genotype** | **Disease reaction of anthracnose races*** | | | | |
| --- | --- | --- | --- | --- | --- | --- |
|  |  | **2047** | **3** | **73** | **87** | **513** |
|  | WB-1255 | 0.5 | 4.0 | 2.0 | 4.4 | 4.0 |
|  | WB-1184 | 3.6 | 5.0 | 5.0 | 1.0 | 5.0 |
|  | WB-206 | 5.0 | 3.0 | 5.0 | 1.4 | 5.0 |
|  | WB-1316 | 3.5 | 0.5 | 5.0 | 5.0 | 3.0 |
|  | WB-1492 | 3.8 | 5.0 | 0.0 | 5.0 | 4.8 |
|  | WB-1181 | 0.3 | 4.0 | 3.6 | 5.0 | 5.0 |
|  | WB-1436 | 4.6 | 0.0 | 5.0 | 5.0 | 3.5 |
|  | WB-1446 | 3.6 | 5.0 | 0.0 | H | 3.0 |
|  | WB-1190 | H | H | 4.3 | 5.0 | 0.4 |
|  | WB-1151 | 4.5 | 1.3 | 5.0 | 5.0 | 4.8 |
|  | WB-1171 | 0.0 | 3.1 | 5.0 | 1.2 | 5.0 |
|  | WB-1304 | 4.3 | 0.0 | 5.0 | 0.0 | 3.0 |
|  | WB-1319 | 0.0 | 5.0 | 5.0 | 5.0 | 5.0 |
|  | WB-1137 | 4.8 | 4.6 | 5.0 | 0.6 | 5.0 |
|  | WB-1136 | 4.8 | 4.0 | 0.8 | 5.0 | 4.6 |
|  | WB-1664 | 5.0 | 0.2 | 5.0 | 5.0 | 4.2 |
|  | WB-1634 | 0.1 | 0.0 | 5.0 | 0.0 | 0.1 |
|  | WB-1150 | 5.0 | 0.0 | 5.0 | 5.0 | 2.0 |
|  | WB-1643 | 4.8 | 0.0 | 4.3 | 5.0 | 4.6 |
|  | WB-1131 | 5.0 | H | 5.0 | 0.3 | 4.6 |
|  | WB-1129 | 5.0 | 0.0 | 5.0 | 0.0 | 5.0 |
|  | WB-1177 | 5.0 | 1.2 | 5.0 | 5.0 | 4.1 |
|  | IC-313295 | 4.3 | 2.0 | 4.6 | 5.0 | 4.3 |
|  | 22721 | 5.0 | 0.2 | 5.0 | 5.0 | 5.0 |
|  | IC-285575 | 4.8 | 0.2 | 5.0 | 0.0 | H |
|  | G-22552 | 4.8 | 0.0 | 3.7 | 0.0 | 5.0 |
|  | Jawala | 4.0 | 0.0 | 5.0 | 0.0 | 5.0 |
|  | WB-4564 | 5.0 | 5.0 | 0.0 | 5.0 | 0.5 |
|  | WB-5176 | 5.0 | 5.0 | 0.0 | 4.0 | 5.0 |
|  | WB-6960 | 5.0 | 4.8 | 1.5 | 3.3 | 5.0 |
|  | WB-1282 | 4.1 | 0.8 | 5.0 | 0.0 | 4.5 |
|  | WB-1185 | 4.3 | 5.0 | 5.0 | 3.0 | 0.0 |
|  | WB-901 | 0.0 | 0.5 | 4.8 | 0.5 | 4.6 |
|  | WB-869 | 0.0 | 2.7 | 3.8 | 3.6 | 5.0 |
|  | WB-864 | 5.0 | 0.5 | 5.0 | 5.0 | 5.0 |
|  | WB-832 | 5.0 | 0.2 | 5.0 | 1.0 | 5.0 |
|  | WB-451 | 0.0 | 3.0 | 5.0 | 0.4 | 4.2 |
|  | KRC-5 | 5.0 | 0.0 | 1.2 | 2.6 | 5.0 |
|  | WB-1691 | 5.0 | H | 2.3 | 0.6 | 1.1 |
|  | WB-956 | 4.8 | 4.8 | 1.0 | 5.0 | 5.0 |
|  | WB-1006 | 0.0 | 3.0 | 5.0 | 5.0 | 5.0 |
|  | WB-967 | 0.0 | 0.0 | 0.0 | 0.0 | 0.0 |
|  | WB-507 | 5.0 | 0.6 | 2.0 | 3.0 | 0.8 |
|  | WB-630 | 0.6 | 3.3 | 5.0 | 5.0 | 5.0 |
|  | WB-642 | 5.0 | 0.6 | 5.0 | H | 3.0 |
|  | WB-634 | 5.0 | 1.2 | 4.8 | 2.0 | 4.2 |
|  | WB-650 | 0.3 | 3.6 | 5.0 | 5.0 | 5.0 |
|  | WB-665 | 5.0 | 5.0 | 5.0 | 5.0 | 1.2 |
|  | WB-651 | 3.6 | 3.0 | 2.0 | 5.0 | 5.0 |
|  | WB-716 | 4.8 | 1.1 | 0.0 | 0.5 | 0.6 |
|  | WB-371 | 5.0 | 5.0 | 4.5 | 0.0 | 1.4 |
|  | WB-352 | 0.0 | 0.0 | 3.7 | 3.0 | 5.0 |
|  | WB-335 | 3.1 | 0.5 | 5.0 | 0.0 | 1.0 |
|  | WB-333 | 5.0 | 0.0 | 2.0 | 0.0 | 4.6 |
|  | WB-258 | 5.0 | 0.4 | 1.5 | 2.0 | H |
|  | WB-252 | 0.0 | H | H | H | 5.0 |
|  | WB-115 | 4.2 | 0.8 | 4.0 | 5.0 | H |
|  | WB-243 | 4.6 | 0.0 | 0.0 | 4.0 | 3.0 |
|  | WB-242 | 5.0 | 0.2 | 5.0 | 0.0 | 3.5 |
|  | WB-216 | H | 0.0 | 0.0 | 0.0 | 5.0 |
|  | WB-185 | 5.0 | 0.0 | 1.6 | 2.8 | 5.0 |
|  | WB-191 | 5.0 | 0.0 | 5.0 | 0.0 | 5.0 |
|  | WB-186 | 4.4 | 0.6 | 5.0 | 1.0 | 5.0 |
|  | WB-112 | 5.0 | 5.0 | 1.5 | 3.2 | 5.0 |
|  | PBG-102 | 5.0 | 5.0 | 0.0 | 5.0 | 5.0 |
|  | WB-92 | 5.0 | 0.0 | 5.0 | 0.0 | 1.5 |
|  | WB-83 | 0.0 | 0.2 | 2.6 | 2.8 | 3.8 |
|  | WB-46 | 5.0 | 0.2 | 4.8 | H | 5.0 |
|  | WB-5178 | 3.0 | 0.0 | 5.0 | 5.0 | 5.0 |
|  | WB-1587 | 4.1 | 3.0 | 0.0 | 5.0 | 5.0 |
|  | WB-1690 | 5.0 | 0.0 | 1.8 | 5.0 | 4.6 |
|  | WB-1310 | 0.0 | 0.0 | 5.0 | 4.6 | 4.4 |
|  | GLY-P | 0.0 | 5.0 | 3.0 | 5.0 | 5.0 |
|  | WB-1490 | 5.0 | 5.0 | 5.0 | 2.0 | 5.0 |
|  | PBG-545 | 2.4 | 0.0 | 4.1 | 0.0 | 5.0 |
|  | WB-765 | 3.6 | 0.1 | 4.5 | 3.2 | 0.4 |
|  | 24044C | 4.5 | 0.5 | 0.0 | 3.7 | 0.4 |
|  | WB-373 | 4.5 | 0.3 | 4.8 | 0.0 | 1.6 |
|  | WB-916 | 3.2 | 0.0 | 5.0 | 3.0 | 5.0 |
|  | WB-957 | 5.0 | 0.0 | 5.0 | 5.0 | 5.0 |
|  | 10928 | 5.0 | 0.0 | 3.8 | 0.0 | 5.0 |
|  | WB-1710 | 5.0 | 1.0 | 5.0 | 3.0 | 3.2 |
|  | WB-1670 | 5.0 | 1.0 | 4.0 | 0.0 | 1.2 |
|  | WB-1518 | 4.6 | 0.2 | 4.8 | H | 0.0 |
|  | WB-45 | 5.0 | 0.0 | 5.0 | 5.0 | 1.6 |
|  | WB-1703 | 4.2 | 0.0 | 5.0 | 0.4 | 1.4 |
|  | IC-321181 | 5.0 | 0.5 | 5.0 | 0.0 | 5.0 |
|  | IC-530923 | 5.0 | 0.0 | 5.0 | 1.8 | 2.0 |
|  | WB-487 | 5.0 | 5.0 | 0.1 | 5.0 | 1.3 |
|  | IC-4850 | 4.1 | 5.0 | 1.6 | 0.2 | 3.4 |
|  | WB-1699 | 4.0 | 4.6 | 0.0 | 3.8 | 5.0 |
|  | IC-319825 | 5.0 | 5.0 | 5.0 | 5.0 | 2.8 |
|  | WB-1685 | 5.0 | 5.0 | 2.0 | 5.0 | 5.0 |
|  | WB-228 | 4.3 | 0.2 | 3.7 | 0.6 | 5.0 |
|  | WB-1704 | 0.0 | 0.0 | 0.0 | 4.0 | 5.0 |
|  | WB-955 | 4.8 | 4.8 | 0.5 | 4.0 | 5.0 |
|  | WB-1680 | 4.6 | 5.0 | 2.8 | 0.0 | 0.0 |
|  | WB-934 | 5.0 | H | 4.0 | 0.8 | 5.0 |
|  | WB-1139 | 3.0 | 0.0 | 0.0 | 5.0 | 5.0 |
|  | WB-1496 | 0.0 | 5.0 | 0.0 | 2.0 | 5.0 |
|  | WB-255 | 3.8 | 0.4 | 3.6 | 5.0 | 5.0 |
|  | WB-418 | 4.5 | H | 4.0 | 1.7 | 5.0 |
|  | SSGB-729 | 4.6 | 5.0 | 0.0 | 1.8 | 5.0 |
|  | KBR-63 | 4.2 | 0.0 | 5.0 | 5.0 | 5.0 |
|  | WB-1709 | 3.0 | 0.2 | 5.0 | 5.0 | 5.0 |
|  | WB-1705 | 3.6 | 4.8 | 4.1 | 1.8 | 5.0 |
|  | WB-1693 | H | 3.8 | 0.1 | 1.0 | 5.0 |
|  | KDFD-3 | 5.0 | 5.0 | 5.0 | 0.4 | 5.0 |
|  | Madwan | 2.2 | 5.0 | 5.0 | 5.0 | 5.0 |
|  | GLC-P | 0.0 | 5.0 | 5.0 | 3.3 | 5.0 |
|  | GLM-P | 1.0 | 5.0 | 0.5 | 0.0 | 5.0 |
|  | GLS-L | 1.6 | 5.0 | 2.2 | 5.0 | 5.0 |
|  | WB-1630 | 1.6 | 5.0 | H | 3.8 | 5.0 |
